# Lightweight PCB defect detection method based on SCF-YOLO

**DOI:** 10.1371/journal.pone.0318033

**Published:** 2025-04-07

**Authors:** Yazhou Li, Yuanyuan Wang, Jiange Liu, Kexiao Wu, Hauwa Suieiman Abdullahi, Pinrong Lv, Haiyan Zhang

**Affiliations:** 1 College of Computer and Software Engineering, Huaiyin Institute of Technology, Huaian, China; 2 Huai’an Power Supply Branch, State Grid Jiangsu Electric Power Co., Ltd., Huaian, China; Purdue University, UNITED STATES OF AMERICA

## Abstract

Addressing the issues of large model size and slow detection speed in real-time defect detection in complex scenarios of printed circuit boards (PCBs), this study proposes a new lightweight defect detection model called SCF-YOLO. The aim of SCF-YOLO is to solve the problem of resource limitation in algorithm deployment. SCF-YOLO utilizes the more compact and lightweight MobileNet as the feature extraction network, which effectively reduces the number of model parameters and significantly improves the inference speed. Additionally, the model introduces a learnable weighted feature fusion module in the neck, which enhances the expression of features at multiple scales and different levels, thus improving the focus on key features. Furthermore, a novel SCF module (Synthesis C2f) is proposed to enhance the model’s ability to capture high-level semantic features. During the training process, a combined loss function that combines CIoU and GIoU is used to effectively balance the optimization of different objectives and ensure the precise location of defects. Experimental results demonstrate that compared to the YOLOv8 algorithm, SCF-YOLO reduces the number of parameters by 25% and improves the detection speed by up to 60%. This provides a fast, accurate, and efficient solution for defect detection of PCBs in industrial production.

## Introduction

At present, the primary methods for PCB defect detection include electrical inspection, X-ray inspection, and manual inspection. Electrical inspection utilizes the PCB’s electrical properties to identify manufacturing defects and test analog and digital signal components [1,2]. However, these methods suffer from high manufacturing costs for test fixtures and lengthy programming and debugging times. X-ray inspection detects defects by analyzing the differences in absorption rates of materials but is hindered by slow detection speeds, high failure rates, and long development times [3]. Manual inspection, relying on magnifying glasses [4] or microscopes, involves operators visually inspecting components, solder joints, and printed circuits on PCBs [5]. This method, however, is prone to human error, resulting in misdetections or missed defects.

As the limitations of traditional methods become more evident, deep neural network based models have emerged as a promising alternative for industrial defect detection. Deep neural network models offer advantages such as ease of deployment, reusability, fast detection speed, and high accuracy [6]. However, despite their potential, existing deep learning models for PCB defect detection often face challenges related to large model size and slow inference speed, which are critical in real-time industrial applications. The focus of this study is to address the challenges associated with large model size and slow detection speed in real-time PCB defect detection within complex industrial scenarios.

As deep learning becomes increasingly integrated into various fields, such as the detection of small lesions [7] and lung tumors [8] in the medical field, there is a growing body of research focused on PCB defect detection. This research is particularly concentrated on optimizing model size and improving real-time performance. The MSD-YOLOv5 model [9] achieved a size reduction by combining MobileNet-v3 with CSPDarknet53 and replacing the coupled detection head with a decoupled detection head. This modification allows for its compatibility with mobile devices; however, the model lacks robustness. The focus of YOLOv4-MN3 [10] is to enhance the optimization of YOLOv4 through modifications in its backbone and activation functions. This leads to a reduction in the parameter count from 63.96M to 39.59M and a decrease in computational operations by more than 50%, thus achieving improvements in both memory efficiency and faster inference. However, there is still potential for further optimization in terms of model size and real-time performance. LSYOLO [11] proposed a compressed model with a weight size of only 3.64MB by introducing an Expanded Receptive Field Module and spatial fusion techniques. This led to a 20% reduction in computational requirements while improving accuracy; however, the inference speed was slightly decreased.

For the model to adapt to the actual needs of PCB industrial production, many researchers have made many attempts to lightweight the model. Shilong Zhao [12] and colleagues introduced a TRANS module at the conclusion of the backbone network, serving as a lightweight design to amalgamate multi-level features derived from global context information. This approach aims to enhance small target detection within intricate backgrounds. Additionally, a feature fusion network operating across multiple layers, was devised to seamlessly merge the fine-grained and semantic feature information extracted from the backbone. This enhances the spatial position information between adjacent feature layers. Liu Jinhai [13] and colleagues devised a LightNet model using structural re-parameterization. This approach significantly enhanced the network’s feature extraction capability, decreased the complexity of model reasoning, and implemented a meticulous self-distillation strategy to boost the accuracy of the student model.Nevertheless, these approaches neglect the model’s detection accuracy and real-time performance, failing to investigate strategies for achieving a harmonious equilibrium between model depth and performance.

To enhance the real-time performance of the algorithm, PCB-YOLO [14] achieves a compromise between speed and accuracy by incorporating depthwise separable convolution, resulting in a frame rate of 92.5 FPS (frames per second) and mean Average Precision (mAP) of 95.97%. YOLO-CEA [15] decreases computational operations through optimized Madds, thereby attaining rapid inference speed and robust detection capabilities. These methods offer valuable insights into the optimization of real-time processes and the lightweighting of algorithms. However, the challenge of achieving real-time and lightweight performance while preserving the algorithm’s expressiveness remains a pressing issue.

This paper aims to propose a novel lightweight defect detection model, SCF-YOLO, which seeks to enhance detection speed and reduce model size without compromising detection accuracy for PCB defects in industrial production environments. To tackle these challenges, the paper presents the following contributions. These contributions facilitate efficient and accurate real-time detection of defects in PCBs, providing a solution that is both practical and scalable for industrial applications.

1. By incorporating MobileNet as the backbone network, the model attains a noteworthy decrease in parameters and improves inference speed in comparison to YOLOv8. This renders it highly efficient for real-time industrial applications.
2. The incorporation of the learnable weight fusion module alongside the SCF module facilitates the efficient fusion of superficial, detailed features with profound semantic information. This enhancement greatly improves the model’s capacity to detect small targets and advances the representation of advanced features.
3. The utilization of a combined loss function (CIoU and GIoU) guarantees accurate optimization of target localization, especially for anomalies with subtle or ambiguous characteristics, enhancing the overall detection performance.
4. The proposed model is evaluated using a dataset of PCB defects, which demonstrates its practical value in industrial production. The experimental results indicate that SCF-YOLO achieves an effective balance between speed, accuracy, and model size, providing a reliable solution for real-time defect detection. The lightweight and efficient design of SCF-YOLO enables its potential deployment on edge computing platforms and embedded devices, making it suitable for largescale applications in various industrial environments.

The SCF-YOLO model can be categorized into three fundamental components: Backbone, Neck, and Head. In the Backbone, substitute the YOLOv8 model’s feature extraction network with the compact edition of MobileNetv3. Utilize the lightweight design and faster detection speed of MobileNetv3 to improve the detection speed of this SCF-YOLO model. Then, the introduction of the utilization of learnable weights to understand the significance of various input features [16]. At the same time, a new gain module SCF is added to the Neck part to help the Neck part provide a more expressive feature expression for the final Head part to improve the model performance of the entire head.Thirdly, calculate bounding box regression loss using multiple loss functions (CIoU loss, GIoU loss, Distribution Focal Loss). Utilize the feature of GIoU [17] that is more sensitive to bounding box offsets to adapt to changes in the bounding box during the training process. Combined with the more stable characteristics of CIoU [18] prediction, the model’s generalization ability for targets of different scales is improved. The amalgamation of the three loss functions enhances adaptability, rendering the loss function better suited for defect detection tasks. It consolidates various facets of information, offering a more holistic optimization objective to improve the model’s generalization ability. Finally, the paper verifies the effectiveness and superiority of the SCF-YOLO algorithm on the PCB defect detection task through detailed experiments.

## Overall architecture

MobileNet serves as the underlying network in SCF-YOLO, enabling the extraction of defect features at various scales and levels. This process involves converting crucial details from the input image into feature representations enriched with additional semantic information. In its architecture, MobileNet incorporates a sequence of depthwise separable convolutions to minimize the parameters and computational complexity of the model. MobileNet adopts a linear stacking approach in its network structure, progressively augmenting channel numbers and diminishing feature matrix resolution. This strategy enables efficient extraction of image features, considering both model size and inference speed, without compromising accuracy.

The SCF module adjusts the importance of feature maps in different channels by connecting low-level feature maps and high-level feature maps and by additional branching operations, which makes the module focus on important semantics while accomplishing semantic integration. This approach enables information transfer, fusion and filtering between different levels of feature maps, thus improving performance of the SCF-YOLO algorithm in detecting targets at different scales.

**Fig 1:**
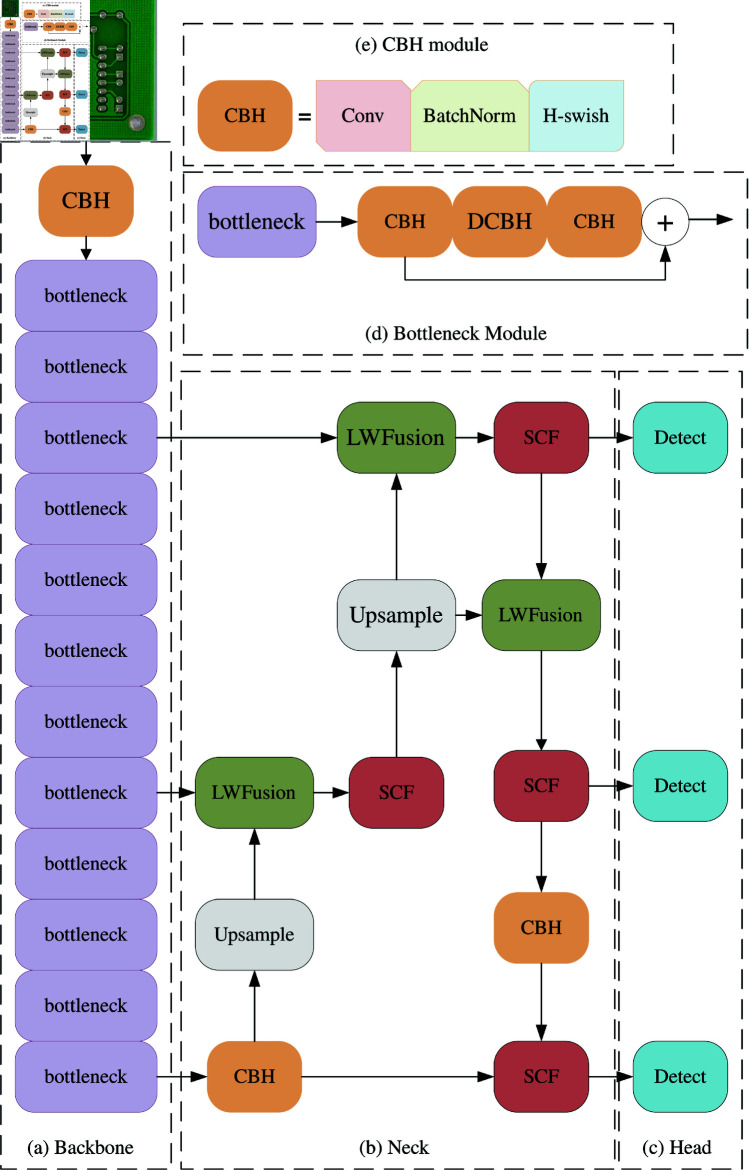
Structural diagram of the overall SCF-YOLO algorithm. (a): Backbone structure diagram. (b): Structure diagram of Neck section (c): Schematic of the Head section. (d): Bottleneck structure diagram. (e): CBH module structure diagram.

The primary semantic features of the input image are extracted in MobileNet, and then the feature pyramid composed of the SCF and CBH modules is integrated with feature fusion to obtain more comprehensive high-level semantic features. The classification and localization tasks are completed by the detection head module. The CBH module consists of a convolutional layer, BatchNorm layer and H-Swish activation layer. First, this design (Fig 1) can use the performance features of MobileNet to lighten the model, and second, it can use the SCF module for feature focusing to make the more valuable semantic features propagate further along the gradient and thus produce positive gains for the final localization and classification work. Lastly, the combination of multiple loss functions is used during model training to guide the optimization of box regression more accurately and achieve the goal of balancing model performance with speed and depth.

### SCF module

**Fig 2:**
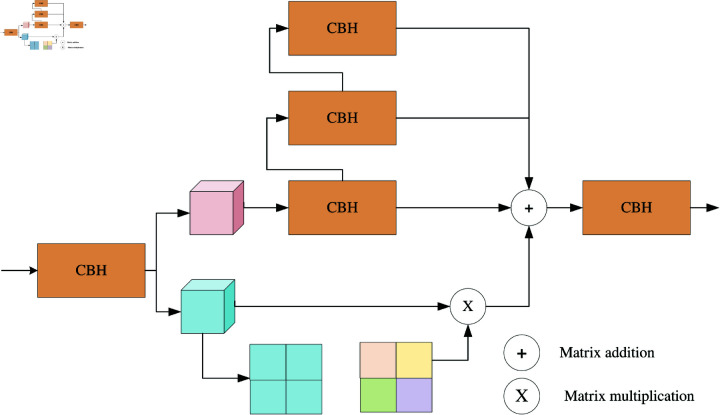
Structural diagram of the SCF module.

By employing a three-branch structure, triplet attention computes attention weights by capturing interdimensional interactions. To address input tensors, the module employs residual transformations, enabling the establishment of interdimensional dependencies and the encoding of interchannel and spatial information which is achieved with minimal computational overhead. The C2f module draws inspiration from the design concepts of both the C3 module and the ELAN [19] module. This amalgamation allows the module to gather more comprehensive gradient flow information while maintaining a lightweight structure. The SCF module is built on the basis of the C2f module with respect to the design idea of Fig 2.

The C2f module empowers the model to identify feature maps with varying scales, amplifies the ability of the model to recognize targets of diverse sizes, and mitigates the extent of feature information loss. However, the module disregard the importance of diverse feature richness across channels. As the number of channels of the feature fusion feature matrix also increases, the SCF module designed in this study allow the model to perform the first step of screening different feature maps in different channels of the original feature map before it performs feature fusion, and then it performs feature fusion to improve the quality of the entire feature enhancement process. On the other hand, this paper focuses only on channel information and does not affect the feature expression of different channels. Thus the number of added parameters is very limited. Selecting the 64-channel SCF module as an example, compared with the C2f module, the number of parameters increases by only 512.

By utilizing the aforementioned operations, the complete network for PCB defect detection not only guarantees exceptional detection performance but also accomplishes the objective of model lightweighting, while introducing only a marginal number of additional parameters. In this case, the performance of the entire model has been greatly improved, and the detection accuracy and mAP(mean average precision) are closer those of to the original model.

### Improving the loss function

In YOLOv8, the loss function includes category loss and position loss. YOLOv8 adopts the same category strategy as RetinaNet, FCOS [20] and other models, uses the sigmoid function to calculate the probability of each category, and uses the BCE loss function to calculate the global category loss, where the category label of the positive sample is the IoU value, and the negative samples are all 0. YOLOv8 divides the position loss into two parts. First, IoU (intersection over union) computation is performed between the predicted bounding box and the target bounding box. Second, the calculation of distribution focal loss promotes the accuracy of boundary box prediction and improves the overall learning efficiency and generalizability.

The CIoU loss is always used, as shown in Eq (1), where, *ρ* is the distance between the centroid of the prediction frame and the real frame, and *c* is the diagonal distance of the smallest outer rectangle. *v* is a correction factor used to further adjust the loss function to adjust the shape and orientation of the target frame; *α* is a weight function. (*w_gt_,h_gt_*) and (*w* , *h*) are the width and height of the target frame and prediction frame, respectively, and the IoU denotes the intersection and concurrency ratio between the real frame and the prediction frame.


LCIoU=1−IoU+ρ2c2+αv,v=4π2(arctan ⁡ wgthgt− arctan ⁡ wh)2,α=v(1−IoU)+v (1)

$$\begin{array}{ccc} Loss=loss_{cls}+loss_{position} & & \end{array}$$ (2)

The overall loss function is shown in Eq (2), where *loss_cls_* represents category loss and *loss_position_* represents position loss, which is composed of *l_CIoU_* and *l_DFL_*. Owing to the ratio nature of the IoU, it remains insensitive to the dimensions of the target object. Nonetheless, in regard to the detection task, the optimization of the bounding box through regression loss and IoU optimization are not entirely interchangeable. Moreover, the *L_n_* norm is sensitive to the object scale, making it unsuitable for directly optimizing the non-overlapping components using the IoU. The position loss calculation in this paper introduces the of GIoU and CIoU [21] techniques. The formula is shown in Eq (3), and the GIoU is shown in Eq (4).


$$\begin{array}{ccc} loss_{position}=l_{CloU}+l_{GloU}+l_{DFL},l_{GloU}=1-GIoU & & \end{array}$$ (3)

$$\begin{array}{ccc} GIoU=IoU-\frac{\left|A_{c}-U\right|}{\left|A_{c}\right|} & & \end{array}$$ (4)

In Eq (4), *A_c_* is denoted as the minimum enclosed area between the predicted box and the actual box; *U* represents the area of intersection between the actual box and the predicted box. *IoU* represents the intersection ratio of the real box and the predicted box. The GIoU metric not only emphasizes the overlapping regions, but also considers the nonoverlapping areas, providing a more comprehensive assessment of the overlap between the predicted box and the actual box.


$$\begin{array}{ccc} Loss=\beta_{1}l_{BCE}+\beta_{2}l_{CIoU}+\beta_{3}l_{GIoU}+\beta_{4}l_{DFL} & & \end{array}$$ (5)

The improved loss function is shown in Eq (5), where $\sum_{i=1}^{4}\beta_{i}=1$, ($\beta_{1}=0.05,\beta_{2}=0.75,\beta_{3}=0.05,\beta_{4}=0.15$). The types of PCB surface defects are classified into just 6 types, and the classification loss converges at a very fast rate during the training process. Therefore assigning a high ratio, but focus on bounding box regression. Compared with *l_CIoU_,l_DFL_* and *l_GIoU_*, which are more auxiliary in nature, the proportion is relatively low. Distribution focal loss can accelerate the convergence process of the model by guiding the model toward better optimal direction at the early stage of training through its unique design. This approach enables the model to achieve not only high detection accuracy but also good robustness. This paper combines the CIoU and GIoU to make their advantages complementary while avoiding their shortcomings. Model robustness can be improved by using a combined loss function.

### Learnable weights fusion module

**Fig 3:**
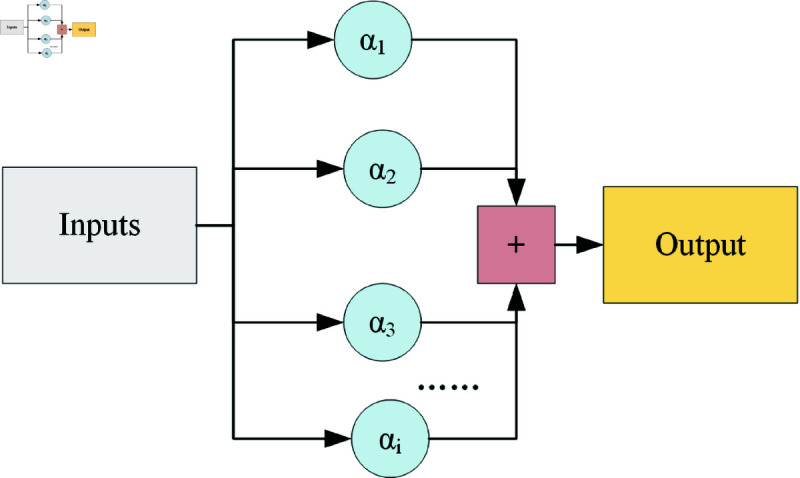
Schematic of Learnable weights fusion module.

In general, the features extracted from different layers of the model differ from each other, with the features extracted from the lower layers favoring the basic primary and local features, while the features gradually become more abstract and advanced as the layers deepen. To make better use of the features at different levels, the learnable weights fusion module is introduced, so that the model can decide by itself to fuse the features at specific layers with specific weights according to the feature distribution of the data. The learnable weights fusion module is shown in Fig 3, where *α_i_* represents the weights of different features.

Compared with traditional feature superposition or feature summation, feature fusion with learnable weights fuses different input features differentially and learns the layers of importance of features. This design possesses an adaptive nature similar to the attention mechanism, giving higher weights to features with high contributions and lower weights to noisy or irrelevant information, which improves the flexibility and generalizability of the model. The final output of the feature fusion approach with learnable weights is shown in Eq (6), where *O* represents the output of the module, *P_i_* represents the input of the module, *α_i_* represents the initial weights and *α_j_* represents the feature weights.


$$\begin{array}{ccc} \text{O}=\frac{\alpha_{\text{i}}{\text{P}}_{\text{i}}}{\underset{\text{j}}{\sum }\alpha_{\text{j}}}(\text{i}=1,2,3,4,...;\text{j}=1,2,3,4,...) & & \end{array}$$ (6)

For each input feature, the weights are within  [ 0 , 1 ] , and the sum of all input feature weights is 1. Since there are no other additional operations affecting the feature fusion, this method of feature fusion does not add extra hyperparameters or affect the inference speed of the model. During training, the optimization algorithm adjusts these weights so that the model is able to dynamically “pick" the combination of features that are most critical to the task at hand. This helps to reduce the impact of irrelevant or redundant information and improves the efficiency and accuracy of the model.

## Experiment

### Data preprocessing

**Fig 4:**
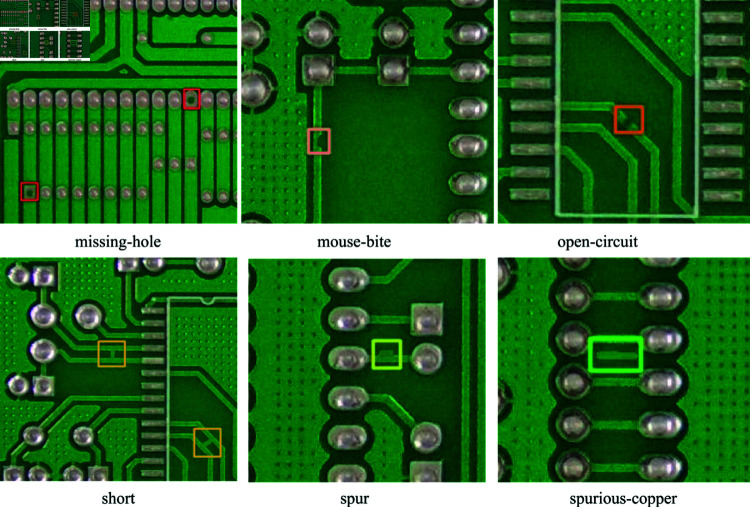
Diagram of 6 common types of PCB defect displays.

**Fig 5:**
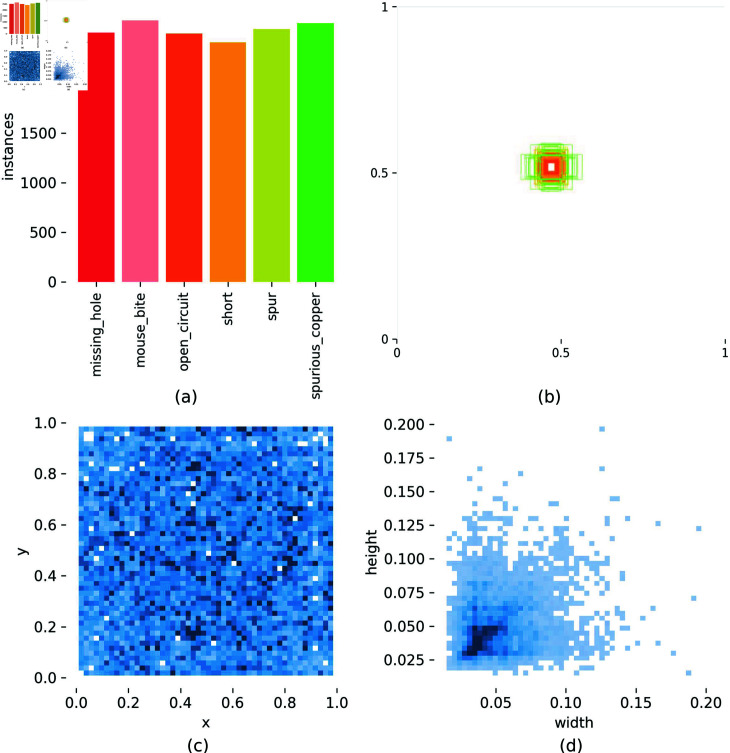
(a): Sample statistical graphs of different types of defects. (b): Number of targets and size distribution. (c): position of the center point of the label relative to the whole image. (d): height-to-width ratio of the target within the image, relative to the overall image dimensions.

Peking University Human—Computer Interaction Open Laboratory released the PCB defect dataset PKU-Market-PCB (https://robotics.pkusz.edu.cn/resources/dataset). The dataset contains 10,668 defects of 6 types: missing-holes, mouth-bite, open-circuit, short, spur, and spurious-copper (Fig 4). The resolution images and corresponding labels, and the specific label distributions are shown in Fig 5. The experiment in this paper draws on the practice of TDD-net [22] to crop the dataset into subimages with a resolution of 320 × 320. To retain the complete defect information as much as possible, each sub-image retains adjacent subimages with a resolution of 10 characteristics of the graph. The self-constructed dataset is partitioned into training, validation, and testing sets with a ratio of 7 : 1 : 2.

In Fig 5(a), the samples of each category are relatively evenly distributed. To enhance the generalizability and robustness of the model, the images undergo random flipping operations, both vertically and horizontally, with probabilities of 50% and 20%, respectively. The images are also randomly enlarged or reduced by 10%. There is a 30% probability of applying MaxUp [23] data augmentation to the images. The objective of employing these data augmentation techniques is to augment the sample diversity by introducing random transformations and expanding the dataset. This approach improves the ability of the model to extract features in complex environments, and allow the entire model to adapt to these environments.

As shown in Fig 5(b) and 5(d), it shows that the target objects in the images are relatively small and that their scales are concentrated. This can lead to blurry contours and less prominent features of the targets. In Fig 5(c), the center points of the targets are uniformly distributed throughout the entire image, rather than being concentrated in specific locations. Therefore, detecting the defective targets in this dataset requires considering more global features to determine the position of the target before the detailed features.

### Experimental equipment and hyperparameter settings

The equipment used in this experiment consists of two NVIDIA A40 model graphics cards. The video memory of a single card is 48G, the single precision is 37.42 TFLOPS, and semiprecision is 149.7 Tensor TFLOPS; To prevent overfitting, the entire experimental process is optimized using the AdamW controller, the momentum to 0.9735, warmup momentum is 0.812, the weight decay is 0.00005, and the nominal batch size is 128. Moreover, in accordance with the experience of many senior researchers, the learning rate is 0.01 and the learning rate attenuation is 0.1.

Following each training session, this study examines the weights acquired through training on the validation set to assess the model’s performance. Throughout the training process, the weights demonstrating the best performance are utilized for the subsequent round of training. In the last 10 rounds of training, no data enhancement operations are performed to help the model better adapt to complex and changeable training samples, thereby increasing the robustness of the model. To avoid contingency of the experimental dataset and improve the persuasiveness of the experiment, three random seeds (0, 3407, 114514) for the experiment, and the experimental results were averaged.

### Backbone network selection

To obtain a lightweight backbone network suitable for PCB defect detection from many networks, the YOLO model serves as the framework in this paper, where the backbone network of the YOLO model is substituted with alternative networks to assess the parameter count, speed of floating point operations, and total number of model layers. In the experiment, only different backbone networks were replaced, and the neck part, decoupling detection head, and other parts did not change. The results are shown in Table 1.

**Table 1:** Comparison of parameters of different backbone networks.

Model	Param(M)	Layers	GFLOPS
YOLO	3.15	225	8.9
MobileNet_small	1.47	357	4.5
MobileNet_large	2.81	398	6.5
Convnext [24]	3.21	301	9.4
Efficientnet [25]	7.39	480	1 4

**Fig 6:**
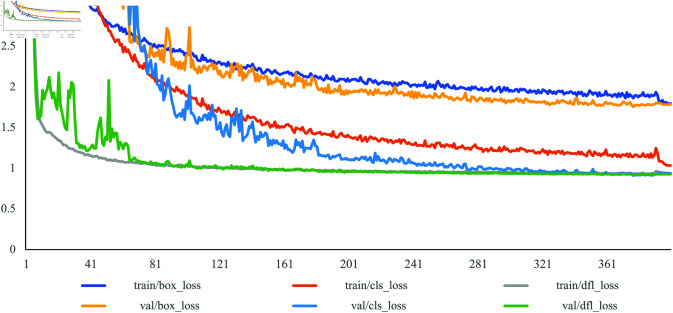
Statistical chart of various loss changes during training.

As shown in Table 1. with MobileNet as the backbone network, the small version of MobileNet has a significantly smaller number of parameters than does the backbone network of the YOLO model. Despite not being the most performant in terms of the GFLOPS, the small version of MobileNet excels in having a remarkably low parameter count, which makes it well-suited for constructing a lightweight PCB defect detection model that meets the desired requirements. Although the GFLOPS and number of layers of the entire model when the Convnext is utilized as the backbone network resemble to YOLO, the parameter count in Convnext is higher and cannot achieve the purpose of lightweighting the entire network. Therefore, in this study Mobilenet is selected as the backbone network of the PCB defect detection model to perform the first step of lightweighting the original model.

### Comparative experiments

As shown in Fig 6, it shows the progression of the three significant loss values, namely regression loss (box_loss), classification loss (cls_loss), and confidence loss (dfl_loss), as obtained by each iteration of the improved model throughout the training process. As the training time increases, the loss value of the model continues to decrease. The regression loss of SCF-YOLO decreases slightly slower than the other losses do, but the decrease process tends to be stable. Given the limited number of PCB defect types, which amounts to only six categories, the classification loss of SCF-YOLO has entered a stable stage in the early stage of training. Subsequent model iterations are used mainly to locate the locations of defects and improve confidence. With an increase in the number of iterations, the curves of the three loss values gradually coverge. When the number of trainings reaches approximately 360, each curve tends to be stable.

In certain scenarios, such as automated inspection of high-speed production lines, the real-time demands for PCB defect detection are very high. Nevertheless, the existing defect detection models are hindered by their high computational demands, rendering them inadequate for real-time detection in a production environment. To verify the real-time performance of the SCF-YOLO model, this study conducted a real-time comparison experiment with the YOLO model. To allow the model to converge during the experiment, the number of epochs 400. Table 2 displays the experimental results.

**Table 2:** Comparison table of real-time performance between SCF-YOLO model and original model.

Model	Param (M)	GFLOPS	FPS (frame/s)
YOLO	3.01	8.2	1000
SCF-YOLO	2.25 (–25%)	6.54	1600

By Table 2, it that the proposed SCF-YOLO model in this paper results in a 25% reduction in the number of parameters compared with the YOLO model. Furthermore, the FPS increased by 60%. The real-time performance of the SCF-YOLO model is better than of the YOLO model. Since the SCF-YOLO model has fewer parameters and higher real-time performance, it is more capable of PCB defect detection in certain automated production scenarios, and can also meet production scenarios with high detection speed requirements.

To investigate the influence of various positioning loss functions on the overall performance of the model, this study conducted a comparison experiment with different positioning losses and compared the main parameters of the model performance using the GIoU and the joint loss function while keeping the experimental conditions unchanged, as shown in Table 3.

**Table 3:** Performance comparison table of models with different loss functions.

Loss Function	Precision (%)	Recall (%)	mAP@0.5 (%)
CIoU	90.3	80.1	55
GIoU	88.4	78.4	86.5
CIoU + GIoU	91.59	90.2	92.9

Table 3 shows that by testing different IoUs on the performance of this model under the PKU-Market-PCB dataset, the joint loss function outperforms other positioning loss functions, exhibiting a 1.6% improvement in precision over the CIoU. Owing to the small size of PCB image targets and subtle changes in defect size and shape, low-quality images are inevitably included in the training data. The GIoU loss function tends to degrade into an IoU loss when the intersection between the predicted position and the ground truth position is minimal.

On the other hand, CoU loss incorporates the aspect ratio of the predicted box to the ground truth box, enabling the loss function to prioritize shape characteristics. This approach compensates for the limitations of the GIoU in terms of intersection measurement. However, the CIoU increases the computational cost of training during complex calculations and slow down the training time. However, the GIoU introduces a minimum closed rectangle to ensure a relatively stable the training process. The hybrid loss function reduces the geometric dimension penalty when the predicted frame and the target frame substantial overlap, indicating strong alignment between them, and thereby improving the generalize ability of the model. Therefore, introducing a hybrid loss function into this model is reasonable.

The main task of PCB defect detection is to detect whether a PCB board contains defects that prevent it from completing its important task of carrying other integrated circuit components. Hence, the fundamental aspect of defect detection lies in its accuracy, which refers to the ability of the model to successfully accomplish the task of identifying defects with precision and optimal performance. The article conducts detailed performance comparison experiments on the SCF-YOLO model in the performance comparison section to verify the industrial usability of this model in terms of performance. Table 4 shows the experimental outcomes. Compared with YOLO, the SCF-YOLO model result in a lower depth and width because of its reduced number of parameters. Consequently, its feature extraction capabilities are relatively weaker, resulting in a slightly inferior overall performance compared to that of the YOLO model. Although the algorithm is less expressive than YOLO, it still has a decent performance for PCB surface defects. The SCF-YOLO model achieves a detection accuracy approaching 95%, coupled with a detection recall rate exceeding 90%. The model performance is maximized through a significant reduction in the number of parameters.

**Table 4:** Performance comparison table between SCF-YOLO model and original model.

Model	Precision (%)	Recall (%)	mAP50 (%)	F-score
YOLO	96.47 ± 0.2	96.49 ± 0.13	96.68 ± 0.16	0.96
SCF-YOLO	91.59 ± 0.12	90.16 ± 0.17	92.90 ± 0.1	0.94

In the automated construction of enterprise PCB defect detection, accuracy and speed are two key indicators. This paper proposes that the SCF-YOLO model has excellent performance in both aspects and provides enterprises with efficient and feasible technical solutions. Although they are lightweight defect detection models, they are still able to maintain high detection accuracy. Using the lightweight model SCF-YOLO proposed in this paper, companies can greatly increase the detection speed and achieve fast and efficient defect detection without sacrificing accuracy. By leveraging these advancements, enterprises can effectively shorten production cycles, increasing production efficiency, reduce costs, and ultimately bolster their competitiveness in the market.

**Fig 7:**
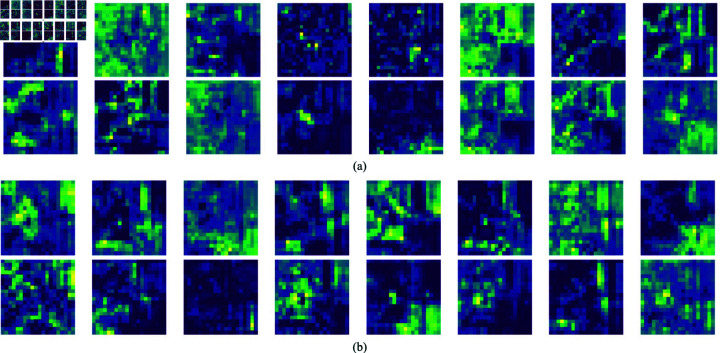
Comparison of the semantic features of the C2f module and SCF module; (a) Semantic feature map for C2f Module; (b) Semantic feature map of the SCF module.

To further amplify the interaction capability of contextual information among feature maps across different channels subsequent to feature fusion, this paper introduces the SCF module at the back of each learnable weight fusion module. The SCF module further improves the quality and quantity of valid information in feature maps in different channels after heavy parameter fusion. In Fig 7, the effective information in layer 12 of the YOLOv8 model is significantly less than that in layer 15 of SCF-YOLO, and the semantic information in each feature map is also relatively vague. Conversely, the SCF module of SCF-YOLO enables the model to obtain more global high-latitude semantic information.

### Category detection results

SCF-YOLO model in the training set until the model converges. After each round of training, the results of the round of experiments are verified, and the images in the test set are tested with the best training weights. Table 5 presents the obtained results.

**Table 5:** SCF-YOLO model’s performances to detect defects of different categories.

Model	Missing hole (%)	Mouse bite (%)	Open circuit (%)	Short (%)	Spur (%)	Spurious copper (%)
Precision	97.9 ± 0.2	87.1	91.3	94	91.5	89.7
Recall	98.9	88 ± 0.1	82.3	95.2 ± 0.04	87.4	83.2 ± 0.8
mAP@0.5	98.5	91.8	91.5 ± 0.9	96.2	89.5 ± 0.6	90.5

Since short-type defects have obvious characteristics and more specific shapes, SCF-YOLO has the best detection effect for this type of defect. The precision, recall, and mAP are higher, at 0.96, 0.89, and 0.94 respectively. Since the features of Spurious copper are more subtle and require a deeper and wider feature extraction network to better extract its subtle feature information, SCF-YOLO has relatively poor recognition ability for this feature, but the model has an IoU of 0.5. The average accuracy reached 90%. Overall, the inspection accuracy of the SCF-YOLO model for each type of defect is nearly 90%.

**Fig 8:**
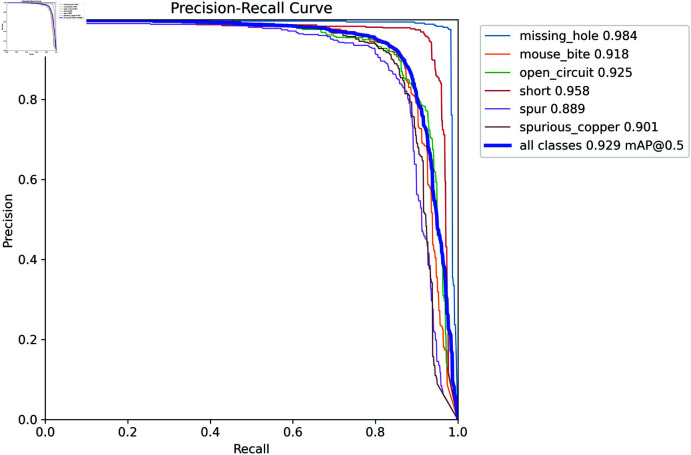
PR curves of SCF-YOLO detection results.

As shown in Fig 8, the Precision-Recall (PR) curves are presented for each category individually, along with the overall PR curve. In the PR curve plot, a curve with an area closer to 1 indicates a stronger performance for that category. The smoothness and small fluctuations in the PR curves reflect good training results. SCF-YOLO demonstrates better detection performance for missing-hole and mouse-bite defects than other types of defects do, which further validates the results presented in Table 5.

**Fig 9:**
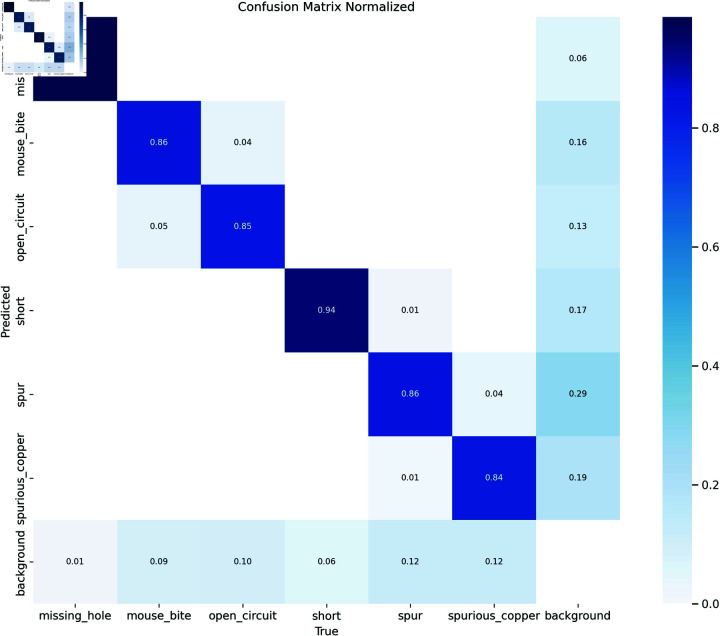
Confusion matrix of SCF-YOLO detection results.

The confusion matrix serves as a critical tool for evaluating the performance of classification models. By comparing the model prediction results of the model with the real labels, various evaluation indicators can be calculated, such as accuracy, recall, precision, and F1 score, can be calculated. The elements in the confusion matrix provide a comprehensive assessment of the model’s performance. As shown in Fig 9, there is a certain lack of ability to extract the two defect features of mouse-bite and spurious-copper. The characteristics of the two defects are similar, and the small defect points lead to unclear contour information about the defects. Therefore, the detection ability of the model is slightly insufficient.

**Fig 10:**
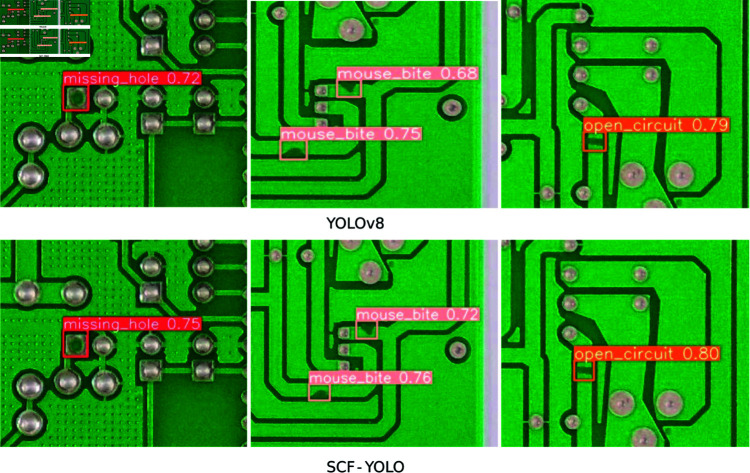
Detection results for missing hole, mouse bite, and open-circuit defects.

**Fig 11:**
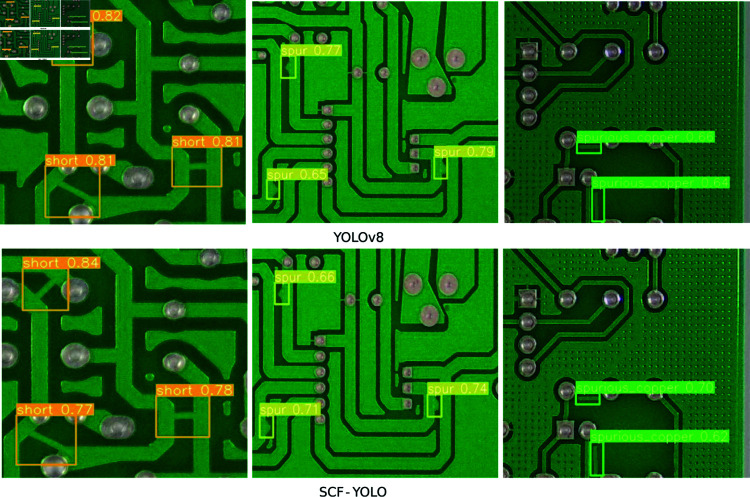
Detection results for short, spur, and spurious-copper defects.

The performance visualization results of the SCF-YOLO lightweight PCB defect detection model proposed in this paper are shown in Figs 10 and 11. Overall, the detection confidence of SCF-YOLO is low, and certain subtle defects are missed. Since the contour information is more subtle for spur-type defects, it is easy to lose the contour information during convolution with other types of defects, resulting in low confidence in the detection model for this type. The depth and depth of the model can subsequently be appropriately improved. Width improves detection confidence. Since the characteristics of short-type defects are very similar to those of normal paths, detection is very difficult, which also result in to low confidence in the SCF-YOLO model. In summary, the SCF-YOLO model successfully fulfills its original design objective by striking a balance between maintaining high detection accuracy and reducing the parameter count of the model, thereby increasing the detection speed of the algorithm.

### Module ablation experiments

To verify which modules in the SCF-YOLO model have the most critical role in the overall performance capability, an ablation experiment based on YOLO is designed. As Table 6 shows, the replacement of the backbone in the SCF-YOLO model resulted in a slight decrease in the overall detection indicators. The precision decreased by 10%, whereas the recall rate experienced an almost 20% decrease. After the introduction of the learnable weight fusion module, the detection accuracy increased by 3.36%, the recall rate increased by 3.81%, and the FPS decreased by 1.7%. Therefore, the learnable weight fusion module has a role in promoting performance improvement of the algorithm as a whole. After the SCF module to the SCF-YOLO model is added, the accuracy and recall rate increased by 1.5% and 3%, respectively, and the detection speed did not decrease but increased by 2%. The SCF module increases the overall detection performance of the model and has a positive role in advancing its capabilities. At the end of the ablation experiment in this paper study, the CIoU loss function is replaced with a loss function that combines the GIoU and CIoU and increases the model width and depth to 1.3 times the original values. According to the replacement results, the overall new performance of the SCF-YOLO model has been slightly improved, and the detection accuracy has increased by 1%. Moreover, the real-time execution capability of the model has decreased slightly. Overall, the changes made in the YOLOv8 model in this study have a positive effect on the challenging task of PCB defect detection.

**Table 6:** Ablation experiments of different modules in the SCF-YOLO model.

Model	MobileNet	Fusion	SECF	Loss Function	Precision (%)	Recall (%)	FPS (frame/s)
YOLOv8	-	-	-	-	96.47	96.49	1000
SCF-YOLO	Y	-	-	-	86.42	77.28	1660
SCF-YOLO	Y	Y	-	-	89.79	81.09	1630
SCF-YOLO	Y	Y	Y	-	91.31	84.13	1670
SCF-YOLO	Y	Y	Y	Y	91.59 ± 0.12	90.16 ± 0.17	1600

### Comparative experiments of different models

To ascertain the efficacy of the model proposed in this article, a comparative experiment was conducted with YOLOv3-tiny, YOLOv5n, YOLOv6n [26], RetinaNet [27] and CenterNet [28], and the parameters, precision, recall, mAP@0.5, and FPS were used as model evaluation indices. An analysis of the experimental results dataset in Table 7, reveals that among all the models considered, SCF-YOLO possesses the fewest number of parameters. The number of parameters is 1% less than that of YOLOv5n. Despite the relatively lower detection accuracy, YOLOv3-tiny outperforms by 1%. Moreover, the model achieves a detection accuracy nearing 95%, thereby fulfilling the requirements for industrial PCB defect detection. The real-time performance is the highest among all the models, and its FPS is 60% higher than that of YOLOv5n, which has the closest model performance.

**Table 7:** Comparative experiments on the performance of different models.

Model	Parameters (M)	Precision (%)	Recall (%)	mAP @0.5 (%)	FPS (frame*s)
YOLOv3tiny	12.1	91.8	81.5	90.2	1500
YOLOv5n	2.5	95.4	93.3	96.3	1000
YOLOv6n	4.2	95.2	92.0	95.4	1000
YOLOv8n	3.1	96.4	96.5	96.7	1000
RetinaNet	7.6	73.0	83.5	97.6	100
CenterNet	7.6	73.2	83.4	96.0	217
SCF-YOLO	2.2	92.5 ± 0.1	90.2 ± 0.1	92.9 ± 0.1	1600

Compared with other single-stage target detection algorithms, the SCF-YOLO model has significant advantages. RetinaNet, despite its innovations in multiscale target detection, is relatively poor in terms of inference efficiency and computational complexity, which is attributed mainly to its use of a focal loss for training, which substantially increases the computational pressure and memory requirements of the model. On the other hand, CenterNet abandons the traditional NMS(nonmaximum suppression) step and instead performs dense prediction at each pixel location, which is a unique design that improves the comprehensiveness of the prediction but inevitably sacrifices a certain inference speed. Especially in scenarios such as PCB defect detection, the high similarity between target defects and background texture features makes the model susceptible to background noise when the target centroid is located, reducing the accuracy and comprehensiveness of the detection results.

**Table 8:** Comparative experiments with typical lightweight objection detection models.

Model	Parameters (M)	Precision	(%) Recall (%)	mAP @0.5 (%)	FPS (frame/s)
YOLOX-nano[42]	0.9	97.6	61.0	53.7	153
YOLOX-tiny	5.0	98.5	67.0	60.9	170
NanoDet-plus[43]	2.4	92.4	57.7	50.4	45
NanoDet-EfficientNet	3.0	93.0	55.9	48.0	59
NanoDet-t	1.2	93.5	58.2	50.9	54
YOLOv5lite-e[44]	0.7	89.0	76.9	83.0	723
YOLOv5lite-s	1.5	87.7	77.4	83.5	635
SCF-YOLO	2.2	92.5	90.2	92.9	1600

To further demonstrate the superiority of the SCF-YOLO model, this paper compares its performance and real-time performance with those of the lightweight target detection models YOLOv5-Lite, YOLOX-nano, and NanoDet. The algorithms in Table 8 all are approximately the same size as SCF-YOLO, but the overall performance of the models is vastly different. The YOLOX and NanoDet series of algorithms have a low recall, even though both achieve a precision greater than 90% precision. This finding indicates that the algorithms identify many error targets with low correctness, resulting in a low mAP@0.5. Compared with YOLOX and NanoDet series algorithms, the YOLOv5lite series of algorithms have improved recall and mAP@0.5. However not only does a large gap exist in the speed of checking, but the performance of the model is also poor, with a difference of 4.8% in accuracy, and 12.8% in recall, compared with SCF-YOLO, which achieves a 12.8% difference in recall, and 9.4% difference in mAP@0.5. The above table, also shows that the YOLOv5-Lite, YOLOX-nano, and NanoDet series models still need further optimization in detecting small targets, whereas SCF-YOLO is not.

In terms of detection speed, NanoDet uses deeper network depth for better performance, increasing the time needed for forward propagation and inference. YOLOX and YOLOv5-lite both use Non-Maximum Suppression, making model detection more time-consuming. SCF-YOLO does not use Non-Maximum Suppression and adopts a lightweight design that focuses more on the balance between model depth and performance, resulting in faster overall detection.

Therefore, the SCF-YOLO model achieves a good balance between model performance and inference speed, and can meet working scenarios with high real-time requirements. In conclusion, the SCF-YOLO model can meet the real-time requirements of industrial production, but also make the model lightweight and reduce the dependence on hardware while ensuring the model performance.

### Robustness experiment of the model

**Fig 12:**
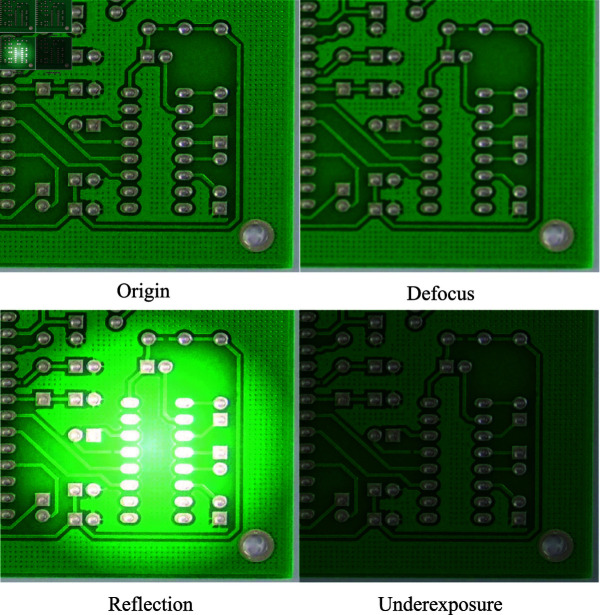
Examples of unexpected situations in real applications.

To verify the robustness of the SCF-YOLO model in practical applications, this paper takes two lighting modes, a coaxial light source and a ring light source, as examples, and considers the possible emergent conditions of the two lighting modes, such as unfocused, reflective and insufficient light, etc. Several pictures are shown in Fig 12, and the corresponding data are collected and augmented to form a new validation set. A sample set containing many special cases is used to validate the robustness of many algorithms.

The SCF-YOLO model is exhaustively compared with the current popular lightweight target detection algorithms in terms of accuracy, comprehensiveness, etc., to prove the advantages of the algorithms and their adaptability to different complex scenarios. The experimental results are shown in Table 9.

**Table 9:** Experimental results on model robustness.

Model	Precision (%)	Recall (%)	mAP @0.5 (%)
YOLOv3-tiny	88.1	78.1	84.8
NanoDet-t	87.5	43.8	52.2
YOLOX-tiny	93.5	61.6	55.2
YOLOv5Lite-e	83.6	68.4	73.7
YOLOv5Lite-s	84.9	69.2	75.4
YOLOv8n	95.0	91.1	94.2
SCF-YOLO	90.8	81.1	90.0

Among the lightweight algorithms (see Table 9), the SCF-YOLO model shows a more balanced performance, and the high precision but low recall of YOLOX-tiny indicate that the algorithm has many misdetections and that the comprehensiveness of the detection is poor, with many misdetections and incorrect detections with high precision. The performance of other lightweight algorithms in this task has a large gap with that of the SCF-YOLO model in terms of both comprehensiveness and accuracy. Owing to the large number of lightweight designs used in this model, the model performance in terms of robustness is still somewhat different from that of the YOLOv8n model. However, compared with the detection performance under normal conditions (see Table 7), the precision is only decreased by 1.7%, and the recall rate is maintained at a high level, which ensures that the algorithm can maintain good detection ability even when it encounters a sudden situation during implementation.

Table 10 clearly expresses the detection ability of the SCF-YOLO model for all types of defects under various conditions. Compared with the normal situation, the detection performance of all classes of defects slightly decreases, with an average decrease in detection accuracy, recall, and mAP@0.5 of 1%,8.1%, and 3.5%, respectively. Episodic situations have a stronger impact on the comprehensiveness of the model, which affects the mAP of the model, with “spur" and “spurious copper" having the strongest impact, both in terms of accuracy and comprehensiveness. Owing to their own small shapes and similar traits, coupled with the interference of external environmental factors, identifying and localizing them accurately creates a great disturbance for the algorithm. Although occasional situations can affect the new performance of the model, the model can be integrated with an external image correction module to provide high-quality inputs to the model, thus ensuring the smooth operation of the actual production. To enhance the detection performance of the algorithm for the categories of “mouse bite" and “spurious copper", this paper employs a Generative Adversarial Network to generate new images. Additionally, it reduces the number of images from other categories to oversample the categories of “mouse bite" and “spurious copper", thus enhancing the depth of the SCF model. The SCF model is trained with 100 epochs of incremental training, building upon the existing training. The experimental results are displayed in Table 11.

**Table 10:** SCF-YOLO algorithm in special cases for each category of detection.

Model	Missing hole (%)	Mouse bite (%)	Open circuit (%)	Short (%)	Spur (%)	Spurious copper (%)
Precision	97.1	86.9	93.1	93.0	88.8	86.5
Recall	92.9	82.7	74.8	86.7	75.6	73.7
mAP@0.5	97.2	85.1	88.9	91.7	86.6	87.1

**Table 11:** Detection results after incremental training.

Model	Missing hole (%)	Mouse bite (%)	Open circuit (%)	Short (%)	Spur (%)	Spurious copper (%)
Precision	96.9	90.4	91.9	93.9	86.8	91.7
Recall	98.3	85.7	83.1	93.6	84.3	83.0
mAP@0.5	92.9	98.4	91.8	92.5	88.9	90.1

Compared with the results presented in Table 10, the model’s performance after incremental training shows improvement for most defects. The augmentation in the depth of the SCF module enhances the model’s capability to capture subtle features and distinguish between similar patterns, particularly evident in defects like “Mouse bites" and “Spurious copper." Nevertheless, with the increased depth of the algorithm, there is a slowdown in inference speed and heightened resource consumption during inference. The augmentation of the dataset enables the algorithm to comprehend more extensive semantic information, encompassing both sophisticated and fundamental aspects. Therefore, this study will explore methods to enhance the accuracy of the model for detecting small targets. These methods include integrating local and global features to enhance small target recognition in intricate backgrounds, introducing extra supervisory signals to facilitate better learning of key features by the model, and flexibly adjusting the confidence threshold of detection results based on scene requirements to enhance detection accuracy and reliability. The goal is to further enhance the performance of the SCF-YOLO algorithm.

In summary, the SCF-YOLO model proposed in this paper is capable of PCB defect detection tasks, has strong robustness to various unexpected situations in the production process, has strong adaptability to the environment, and truly balances the size of the model with the performance of the model.

## Experimental results and analysis

During the experiment in this study, a lightweight PCB inspection model was developed by minimizing the parameter count of the model and increasing the detection speed. Consequently, various feature extraction networks were tested to determine their parameter size, model depth, and computational reasoning capability. To achieve a balance between the size of the model and the depth and reasoning capabilities of the model, MobileNet was selected as the feature extraction network, and further exploration was conducted on this basis. While the reduction in parameters has significantly reduced the weight of the model and resulted in faster performance, it has also caused a notable decrease in the overall model performance (see Table 6). Therefore, this paper introduces the learnable weights fusion module, which uses cross-scale connections and short connections in the same layer to perform multiscale feature fusion to improve the feature expression capabilities as well as the expression capabilities of the entire model. To further improve model performance the idea of ternary attention is used to create a new module SCF based on the C2f module, introducing lightweight channel attention into the C2f module to correct channel characteristics. Two modules are introduced to ensure the detection accuracy of SCF-YOLO, but the recall rate needs to be improved. Hence, in this work, a combined loss function is employed to facilitate more efficient and accurate defect localization by the model. After the experiments, the improvements made to the YOLOv8 model help the model better adapt to the work scenario of PCB defect detection. Moreover, the SCF-YOLO model is lighter than the original model and easier to deploy.

According to the defect detection results of different categories (Table 5), the SCF-YOLO model has room to improve its detection capabilities for mouse-bite and spurious-copper defects. This has much to do with the similarity of defects and the salience of features. First, mouse bite-type defects are very similar to Spur defects in both color and shape. This is an important reason for the poor recognition ability of Mouse-bit e-type defects. Moreover, spurious copper-type defects are very similar to normal routes, which enables the SCF-YOLO model to identify them as normal. Second, PCB defects are minimal, which results in its outline features possibly affecting the model and thus affecting the recognition ability of the model.

To enhance the lightweight nature of the SCF-YOLO algorithm, this study adopts YOLOv8-l as the teacher model for knowledge distillation. However, the experimental outcomes led to a performance decline in SCF-YOLO. This could be due to the possibility that the student model’s capacity to grasp the knowledge of the teacher model adequately was insufficient, resulting in a failure in distillation. In the future, exploration of alternative knowledge distillation techniques such as multilevel distillation [29], hybrid distillation, and adjustment of distillation weights will be pursued to further reduce the algorithm’s weight through the lens of knowledge distillation. Furthermore, this study contemplates structural pruning of existing models to eliminate redundant convolutional layers or channels [30,31], thus minimizing unnecessary parameters. An importance-based pruning algorithm will be utilized to ensure that the eliminated components do not significantly affect model performance. Additionally, weight quantization, which involves converting model weights from floating-point numbers to low-precision integers (e.g., INT8), is identified as an effective approach to decrease memory usage and enhance inference speed. In the future, this research aims to enhance SCF-YOLO’s suitability for resource-constrained industrial applications to offer more efficient and streamlined solutions for industrial production. Additionally, it will actively investigate deploying the algorithm in other domains and broaden its application scenarios.

## Conclusion

In this study, a novel lightweight PCB defect detection model, termed SCF-YOLO, is introduced to effectively address the challenges of large model size and slow detection speed, which are prevalent in real-time defect detection within complex scenarios. By incorporating MobileNet as a feature extraction network, SCF-YOLO substantially diminishes the number of model parameters while enhancing inference speed. Furthermore, the innovative design of the learnable weight fusion module and SCF module successfully integrates shallow fine-grained features with deep semantic information, thereby improving the detection capability for small targets and enhancing advanced feature expression. Additionally, a combined loss function of CIoU and GIoU is employed to achieve precise optimization of target localization. Experimental results demonstrate that, in comparison to YOLOv8, SCF-YOLO reduces the parameter count by 25% and increases detection speed by 60%, thereby facilitating efficient and rapid real-time PCB defect detection without compromising detection accuracy, thus substantiating the model’s practical application value within industrial production.

Although SCF-YOLO performs effectively in PCB defect detection, there remains potential for improvement in fine defect detection accuracy, model lightweighting, robustness, and hardware deployment. Future research will focus on investigating more advanced feature enhancement mechanisms and multi-scale fusion strategies to optimize detection accuracy. Concurrently, we will examine the efficient deployment of the model on embedded devices and edge computing platforms to facilitate its large-scale application in actual production lines. The SCF-YOLO algorithm not only addresses the current key challenges in PCB defect detection but also offers substantial technical support and valuable practical experience for the advancement of related fields. Additionally, it promotes the further expansion of lightweight and efficient real-time inspection technology in industrial applications. To improve the transparency and reproducibility of scientific research, the analysis code for this study has been made available on GitHub (https://github.com/Yishilinyuan/SCF-YOLO) under the Apache 2.0 license.

## Supporting information

S1 File Raw experimental data. This file contains the raw data of the experiment, details of which are described below.Sheet 1 Comparison experiment of different Backbone.Sheet 2 Module ablation experiment.Sheet 3 Robustness comparison experiment.Sheet 4 Loss function comparison experiment.Sheet 5 The performance comparison experiment with the mainstream algorithm.Sheet 6 Specific performance of each category.Sheet 7 Variation curves of various losses during model training and validation.
(XLSX)

S2 File Data enhancement script. (PY)

S3 File Original images. Original images of the images used in the paper.
(ZIP)

S1 Fig Recall confidence curve. (PNG)

S2 Fig Train process. The change curve of main index in the process of model training.
(PNG)

S1 Text Dataset. url: https://www.kaggle.com/datasets/norbertelter/pcb-defect-dataset/data
(DS_Store)
