# Supplementary figures and images for: Lightweight PCB defect detection method based on SCF-YOLO
Source: PLoS One. 2025 Apr 7;20(4):e0318033. doi: 10.1371/journal.pone.0318033 (PMC11975093; doi:10.1371/journal.pone.0318033)

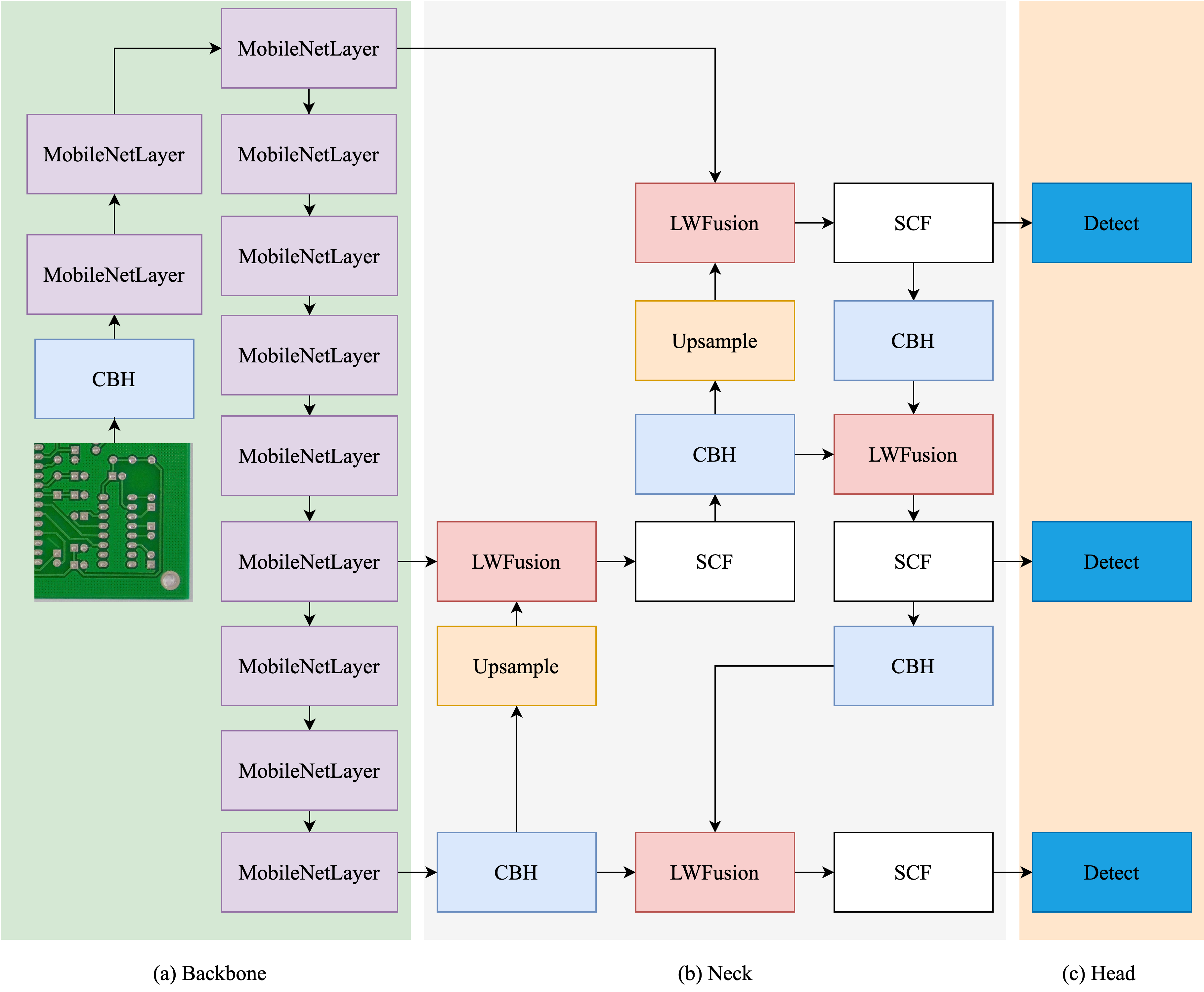

Supplement: S3 File — Original images of the images used in the paper. (ZIP) [file pone.0318033.s003.zip › pone.0318033.s003/fig1.png]

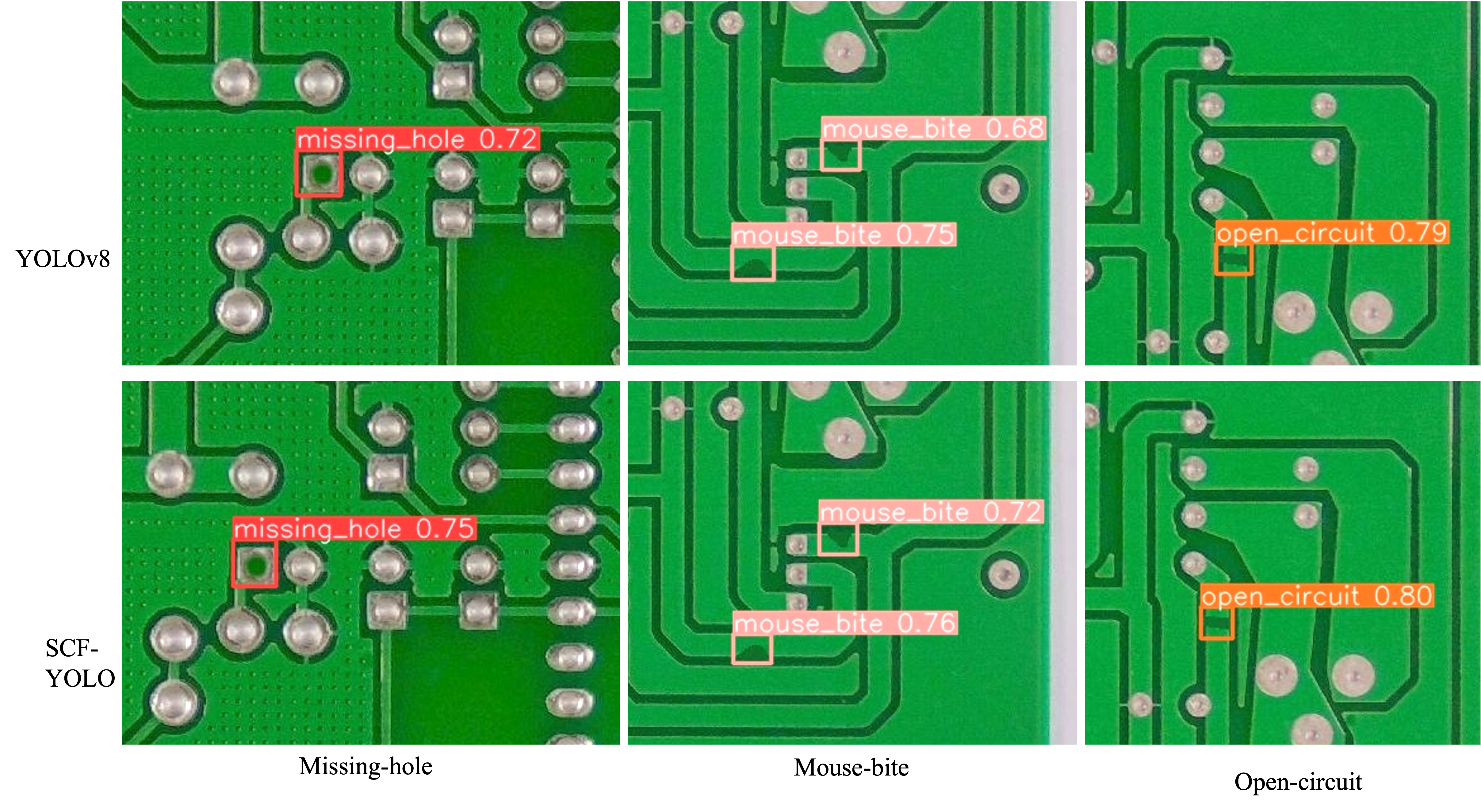

Supplement: S3 File — Original images of the images used in the paper. (ZIP) [file pone.0318033.s003.zip › pone.0318033.s003/fig10.png]

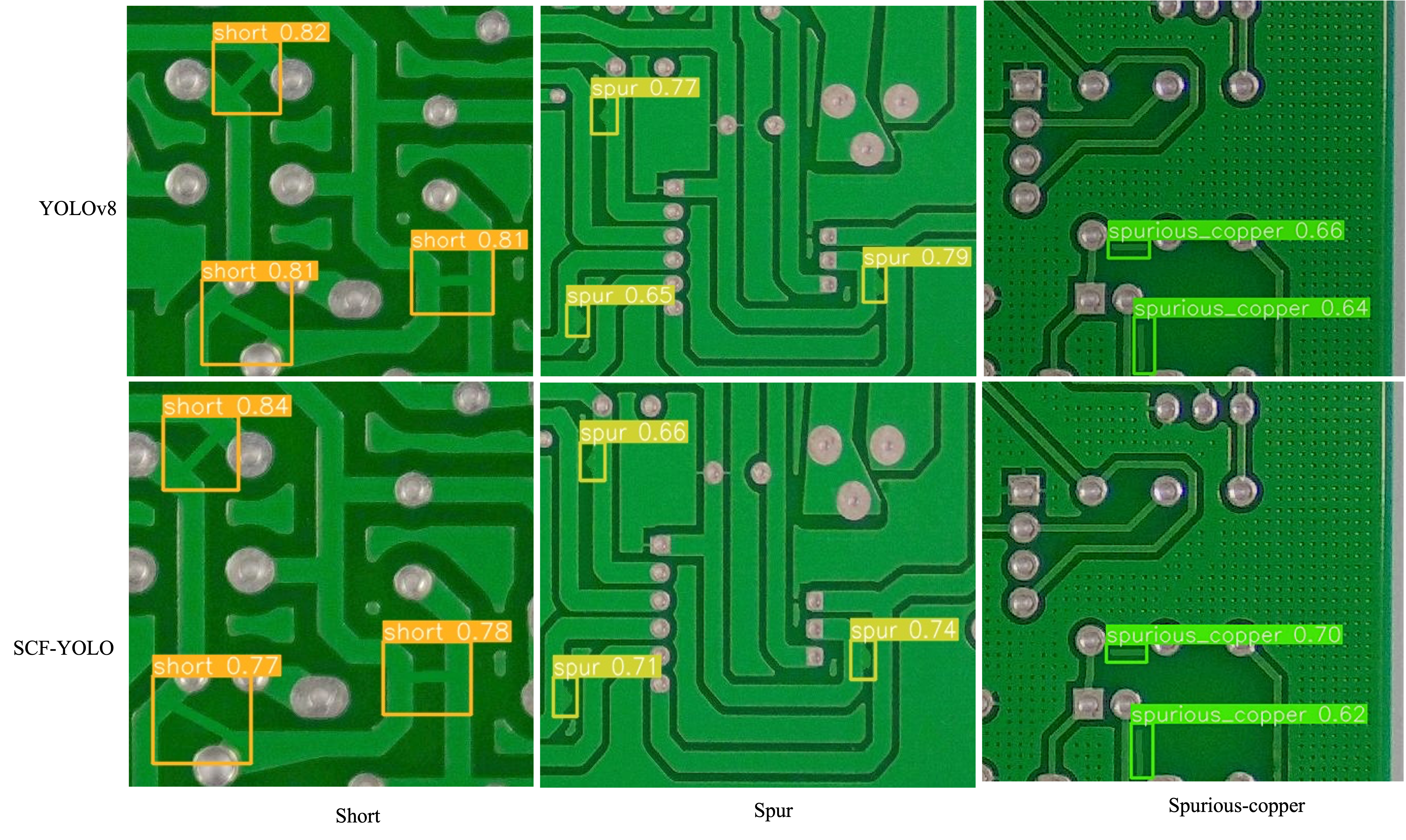

Supplement: S3 File — Original images of the images used in the paper. (ZIP) [file pone.0318033.s003.zip › pone.0318033.s003/fig11.png]

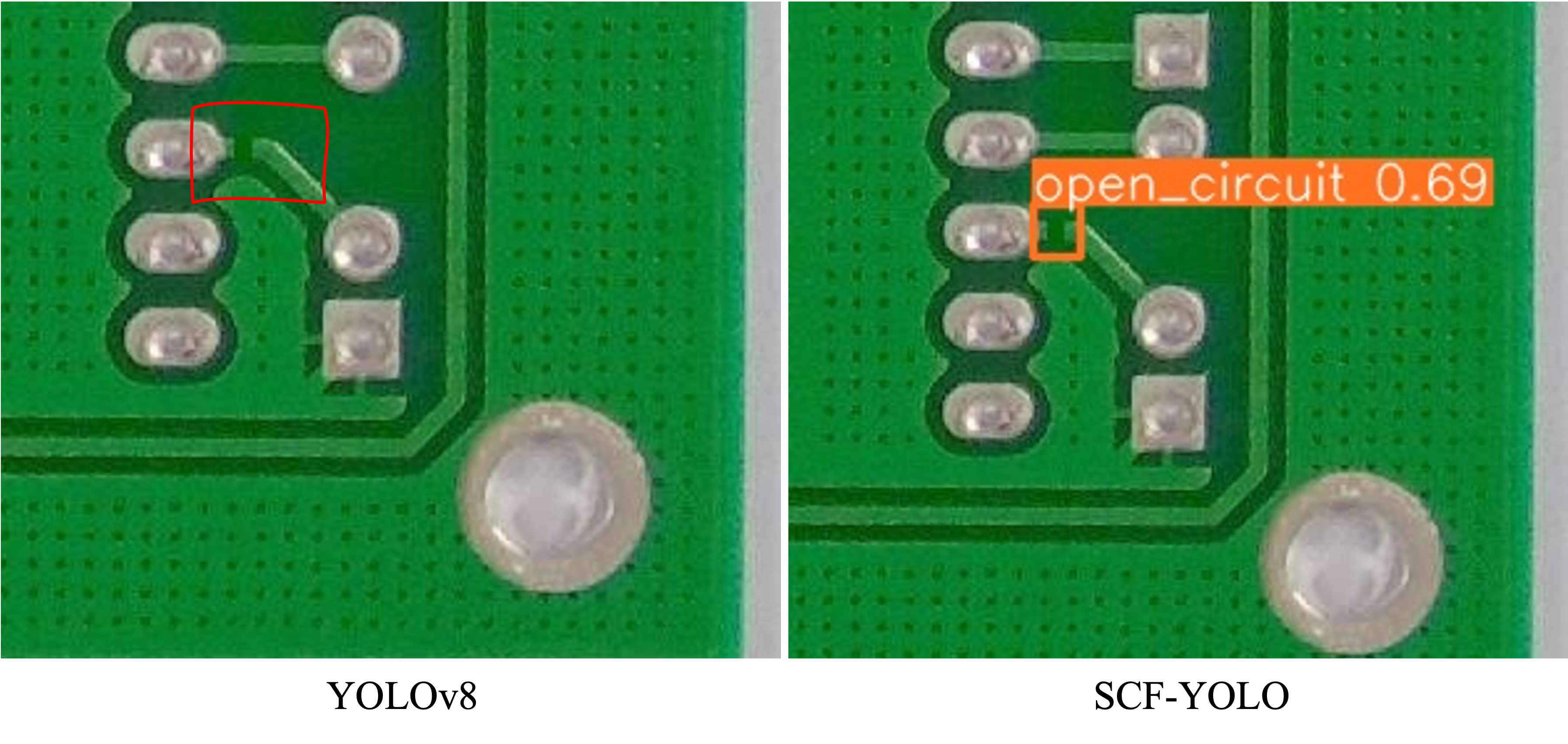

Supplement: S3 File — Original images of the images used in the paper. (ZIP) [file pone.0318033.s003.zip › pone.0318033.s003/fig12.png]

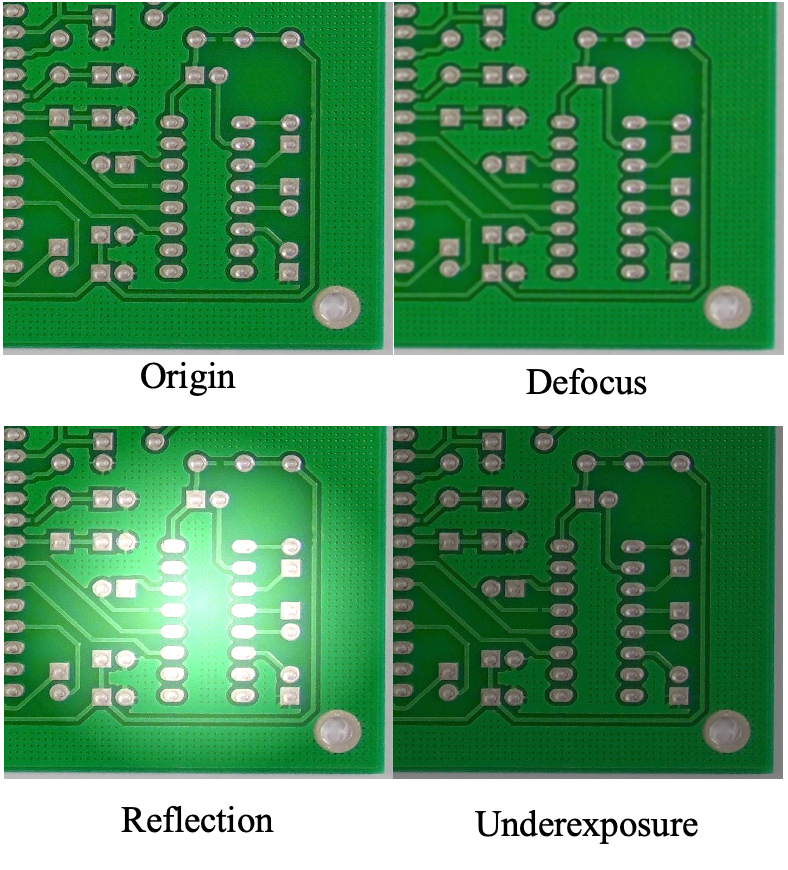

Supplement: S3 File — Original images of the images used in the paper. (ZIP) [file pone.0318033.s003.zip › pone.0318033.s003/fig13.png]

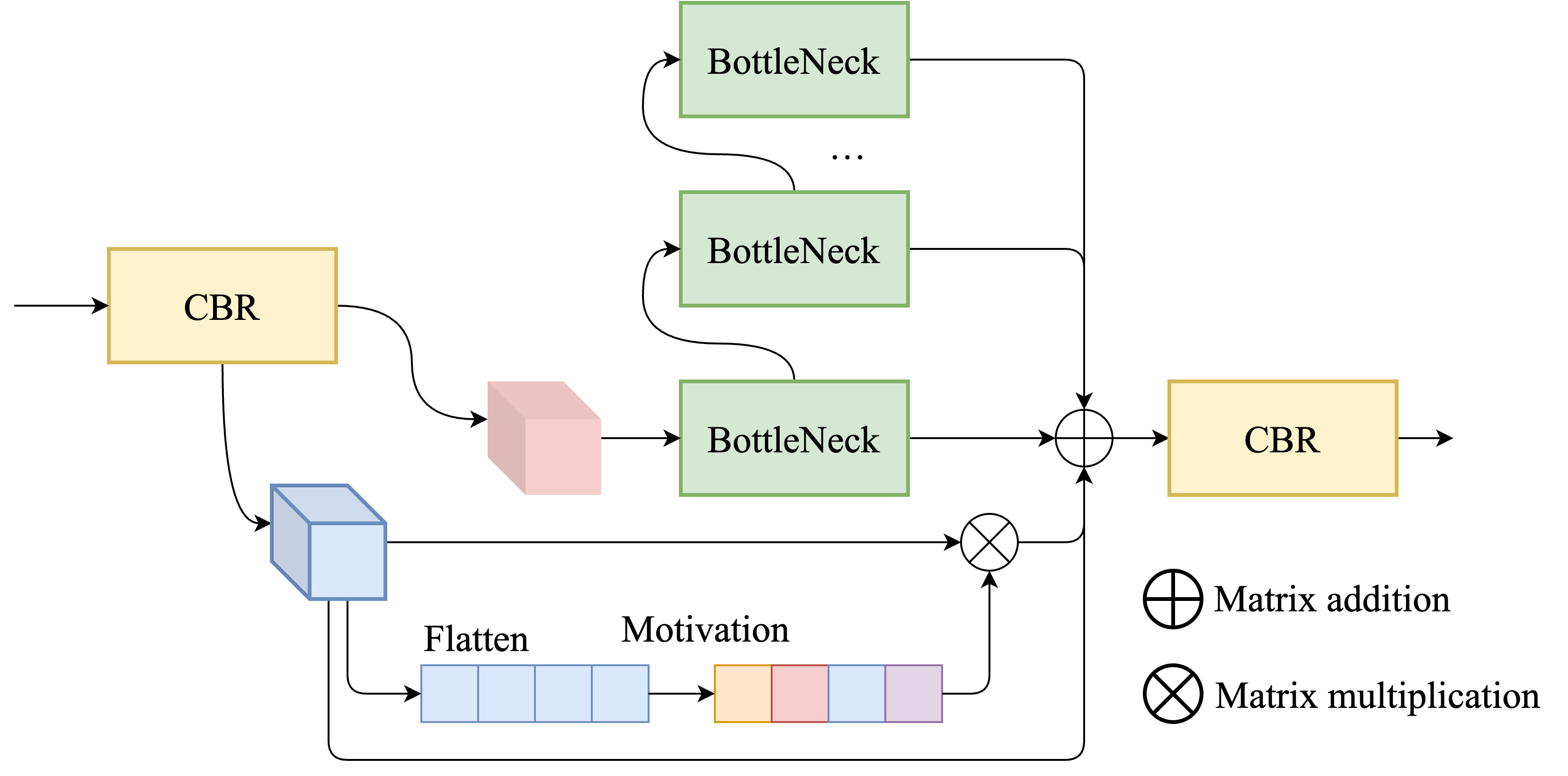

Supplement: S3 File — Original images of the images used in the paper. (ZIP) [file pone.0318033.s003.zip › pone.0318033.s003/fig2.png]

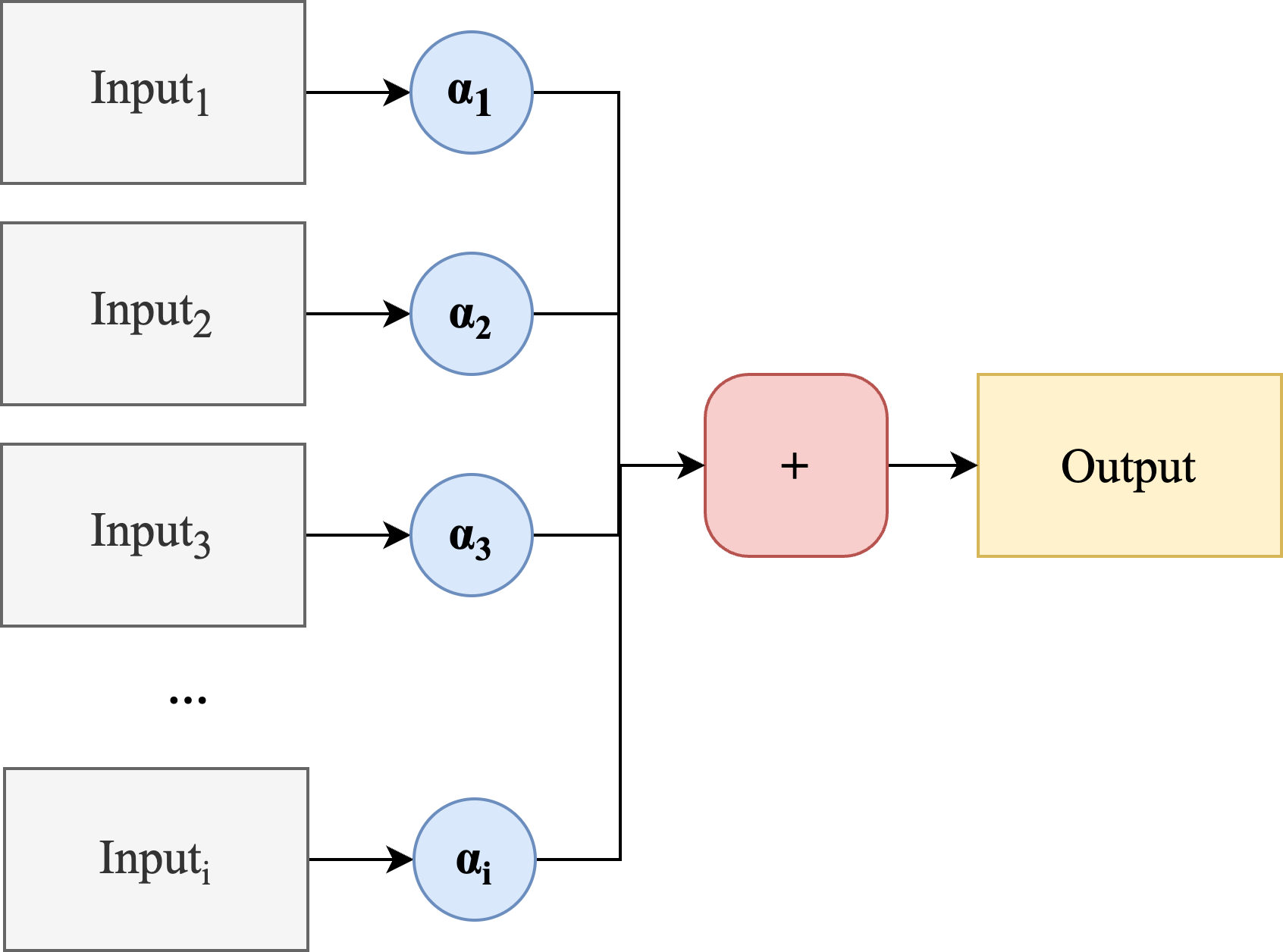

Supplement: S3 File — Original images of the images used in the paper. (ZIP) [file pone.0318033.s003.zip › pone.0318033.s003/fig3.png]

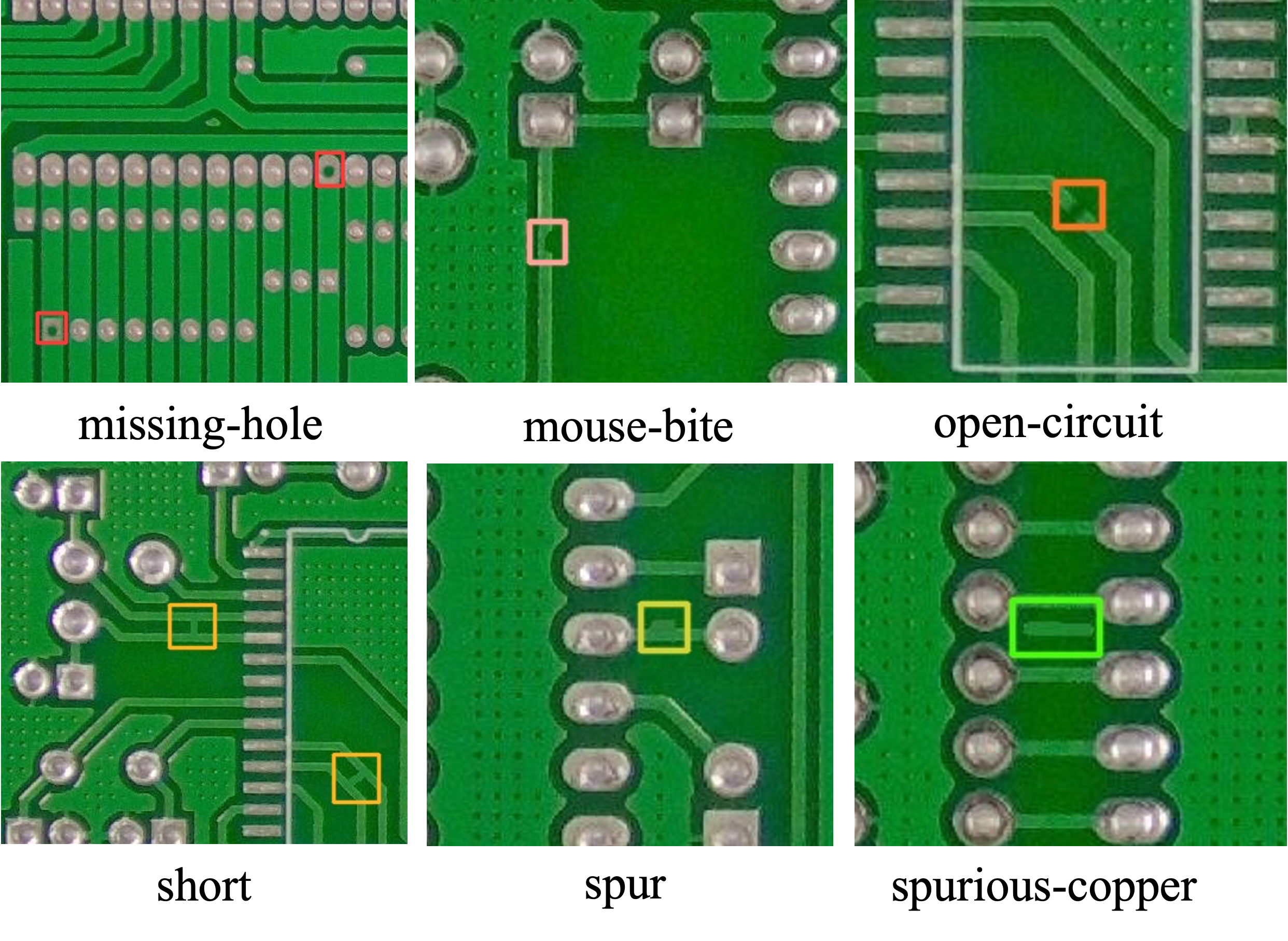

Supplement: S3 File — Original images of the images used in the paper. (ZIP) [file pone.0318033.s003.zip › pone.0318033.s003/fig4.png]

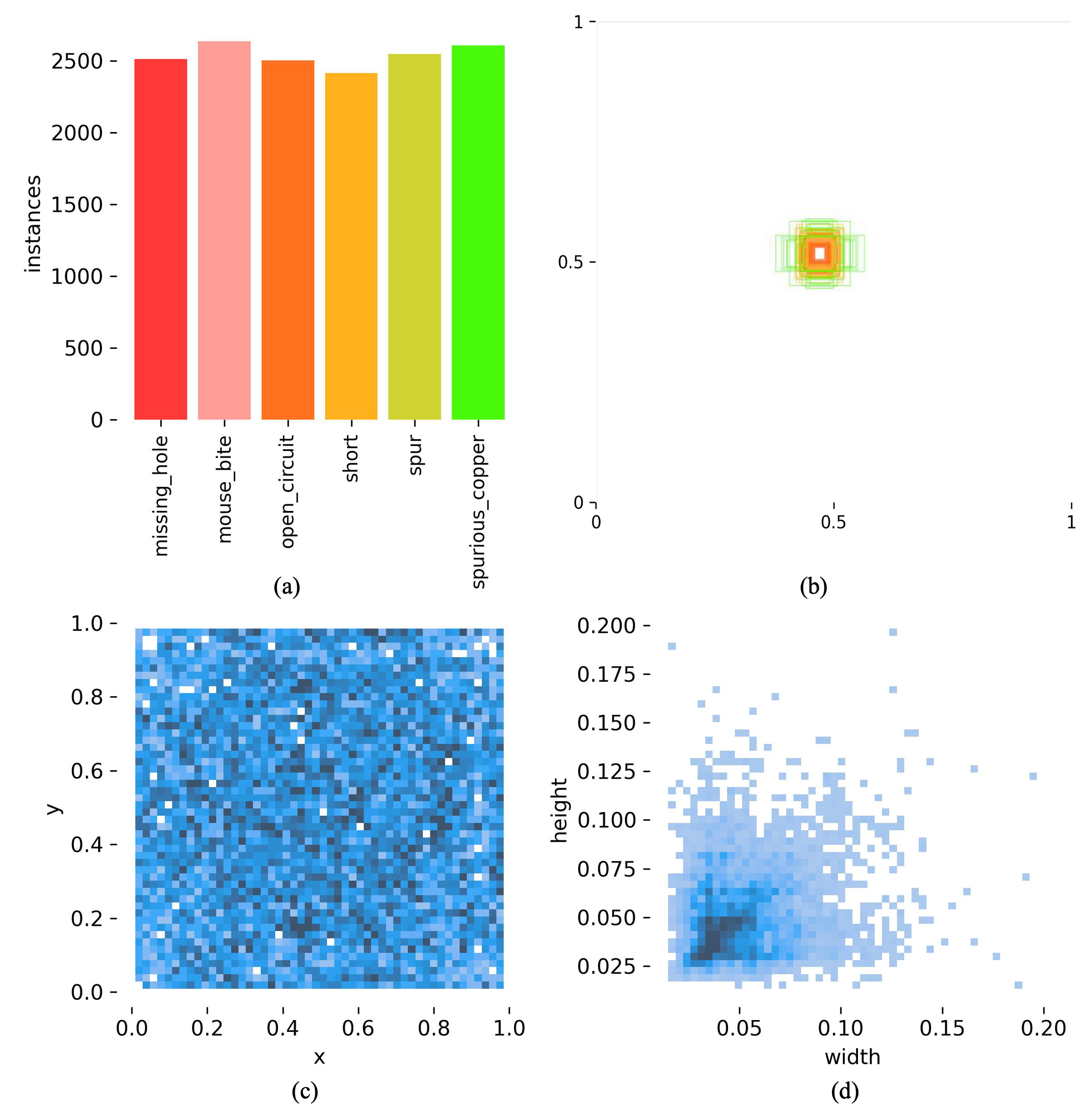

Supplement: S3 File — Original images of the images used in the paper. (ZIP) [file pone.0318033.s003.zip › pone.0318033.s003/fig5.png]

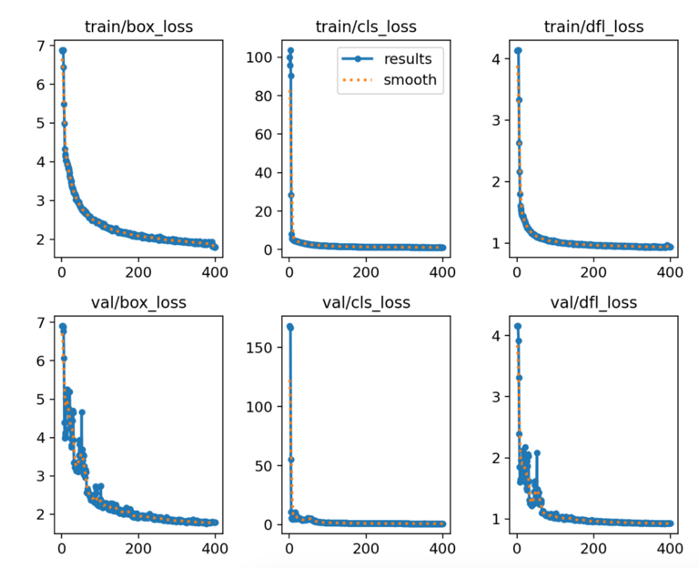

Supplement: S3 File — Original images of the images used in the paper. (ZIP) [file pone.0318033.s003.zip › pone.0318033.s003/fig6.png]

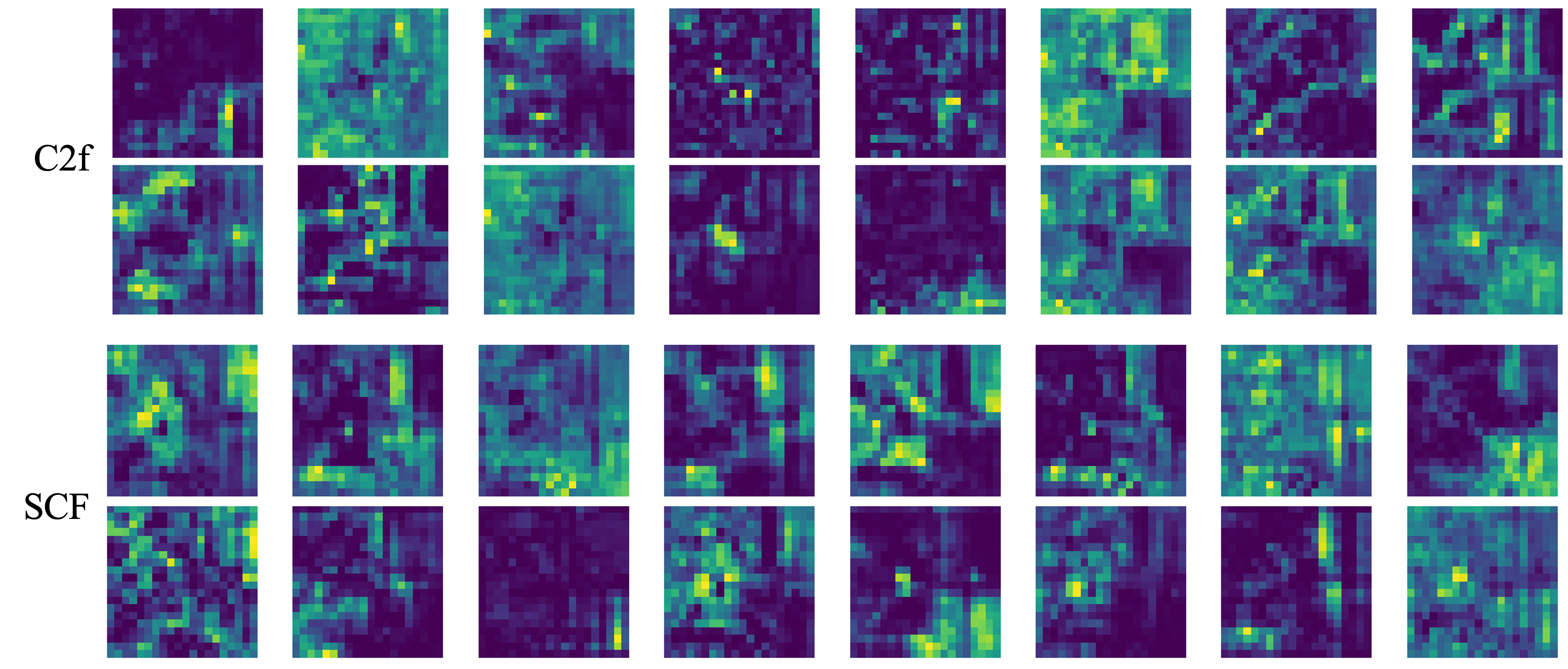

Supplement: S3 File — Original images of the images used in the paper. (ZIP) [file pone.0318033.s003.zip › pone.0318033.s003/fig7.png]

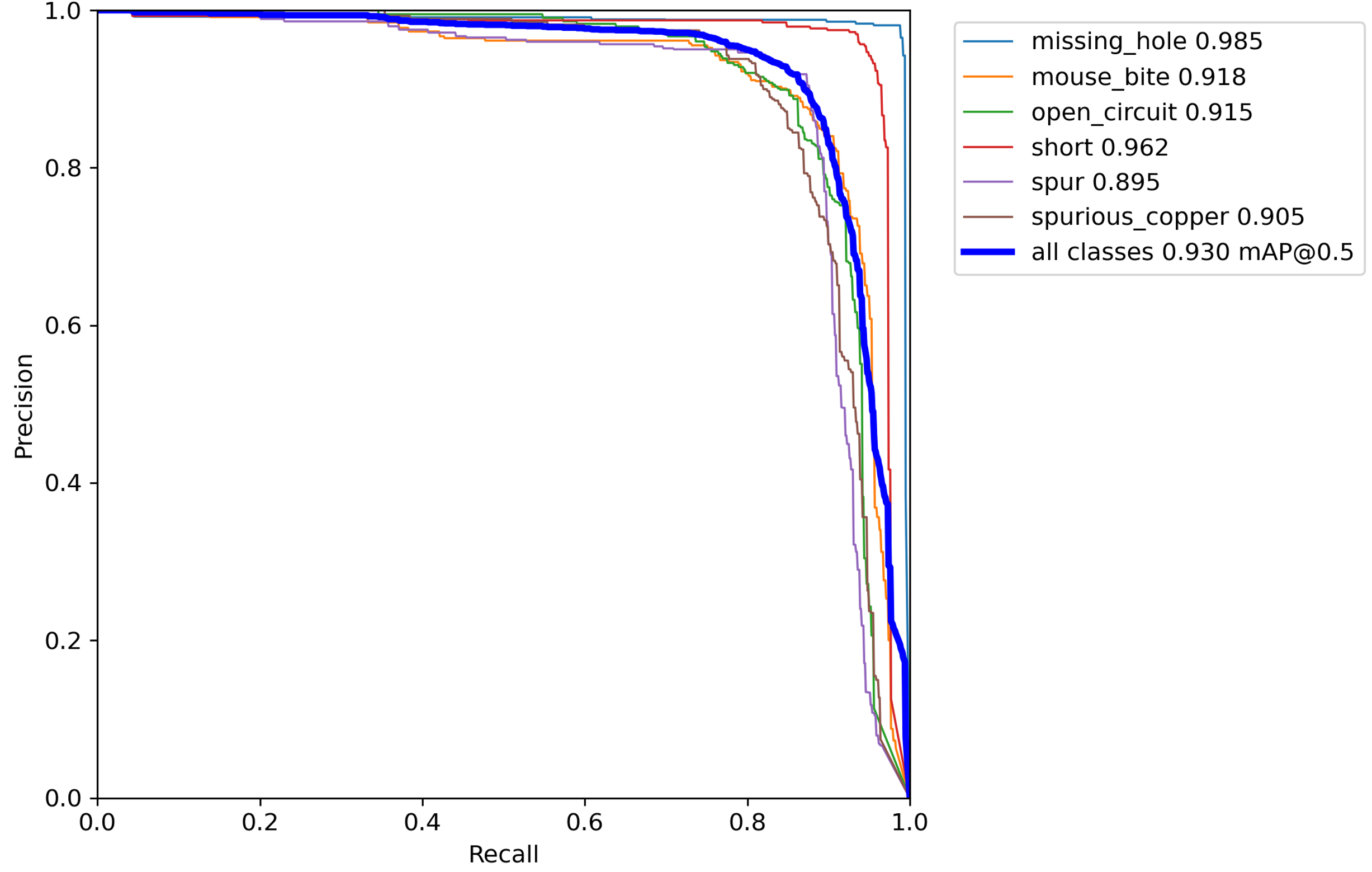

Supplement: S3 File — Original images of the images used in the paper. (ZIP) [file pone.0318033.s003.zip › pone.0318033.s003/fig8.png]

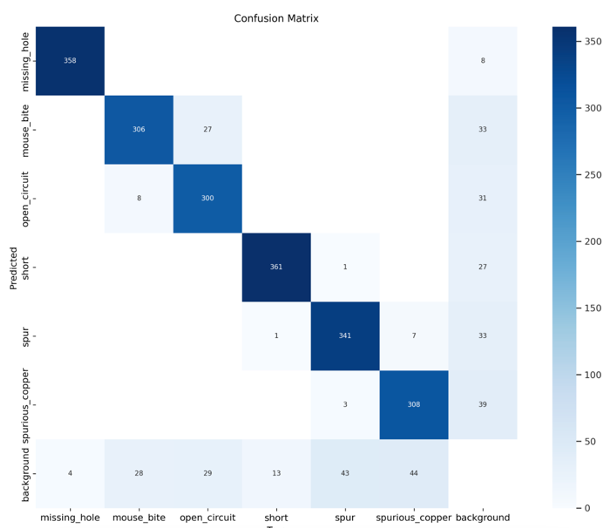

Supplement: S3 File — Original images of the images used in the paper. (ZIP) [file pone.0318033.s003.zip › pone.0318033.s003/fig9.png]

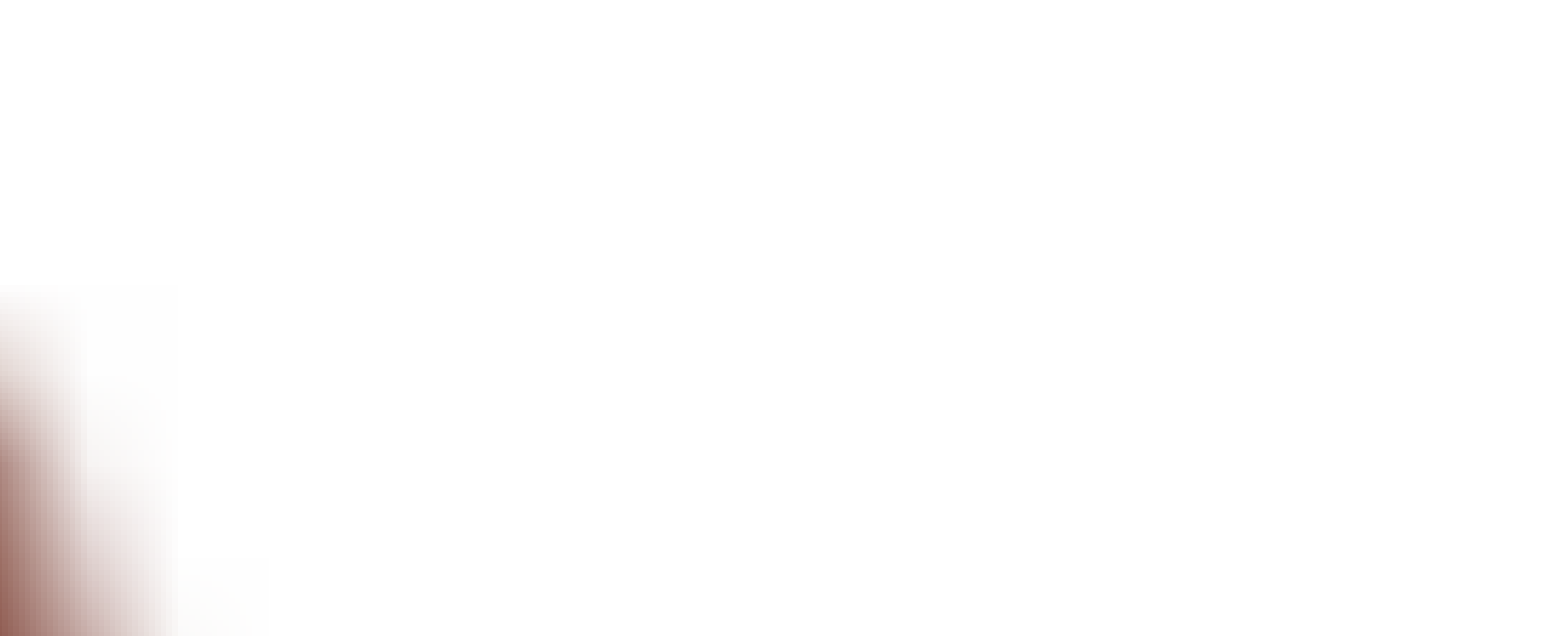

Supplement: S1 Fig — (PNG) [file pone.0318033.s004.png]

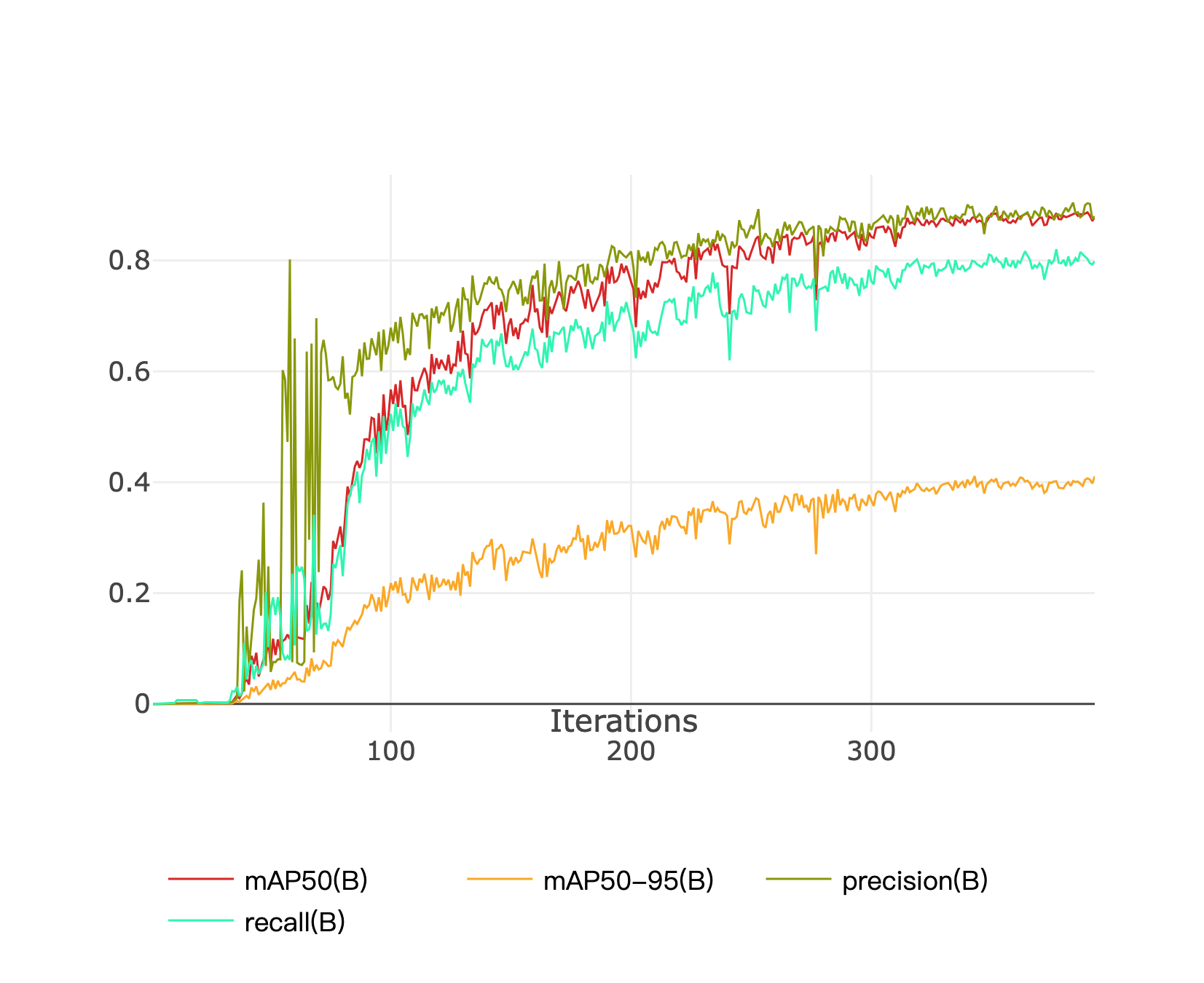

Supplement: S2 Fig — The change curve of main index in the process of model training. (PNG) [file pone.0318033.s005.png]
